# Inhibition of Metalloproteinases-2, -9, and -14 Suppresses Papillary Thyroid Carcinoma Cell Migration and Invasion

**DOI:** 10.3390/ijms26167956

**Published:** 2025-08-18

**Authors:** Domenico Rocco, Vincenzo Marotta, Domenico Palumbo, Mario Vitale

**Affiliations:** Department of Medicine, Surgery and Dentistry, University of Salerno, 84081 Baronissi, Salerno, Italy; vmarotta@unisa.it (V.M.); dopalumbo@unisa.it (D.P.); mavitale@unisa.it (M.V.)

**Keywords:** thyroid cancer, metalloproteinases, NSC405020, gallic acid

## Abstract

Papillary thyroid carcinoma (PTC), while often having a favorable prognosis, can progress to aggressive forms. Matrix metalloproteinases (MMPs) are crucial in extracellular matrix remodeling and are implicated in tumor invasion and metastasis. This study investigated MMP expression and activity in PTC and the efficacy of two selective MMP inhibitors in suppressing PTC cell migration and invasion. The analysis of RNA-seq data from the TCGA-THCA dataset highlighted the overexpression of MMP-14 in PTC, a key upstream activator of several MMPs, including MMP-2 and, indirectly, MMP-9. This elevation correlated with disease status and recurrence risk. Validation in a cell model, using PTC lines (K1 and BCPAP) and non-tumoral thyroid cells (Nthy-ori 3-1), showed markedly increased MMP-14 activity in PTC lines (6-fold in K1; 11-fold in BCPAP). MMP-9 activity was also substantially elevated (386-fold in K1; 131-fold in BCPAP), along with increased MMP-2 activity. We then tested selective inhibitors. NSC405020, an MMP-14 inhibitor, reduced K1 cell migration by 56.52% and invasion by 67.3%. Gallic acid, an MMP-2 and MMP-9 inhibitor, reduced K1 cell migration to 60.3% and invasion to 33.3% relative to the controls. These findings suggest that elevated MMP activity is a hallmark of aggressive PTC, underscoring MMPs’ role in cancer progression. Targeting MMPs, particularly with agents like NSC405020 and gallic acid, presents a promising therapeutic strategy to disrupt PTC tumor progression.

## 1. Introduction

Papillary thyroid carcinoma (PTC) is the predominant form of thyroid cancer, accounting for 80–85% of all thyroid cancer cases [1]. Although a good prognosis in most cases, PTC can evolve into an aggressive and lethal thyroid carcinoma. Nodal metastases in the lateral neck are reported in 27% of patients at presentation, most often originating from tumors in the ipsilateral thyroid lobe [2]. Despite a low mortality around 8%, PTC has a high recurrence rate of 30% [3]. For advanced or radioiodine-refractory thyroid cancer, current therapeutic approaches extend beyond surgery and radioactive iodine, increasingly relying on targeted therapies such as multikinase inhibitors (e.g., sorafenib and lenvatinib) to manage disease progression. However, these treatments often present challenges related to drug resistance and significant side effects, underscoring the continuous need for novel, more effective, and less toxic therapeutic strategies [4]. A deeper understanding of the molecular mechanisms underlying PTC progression is crucial for the identification of accurate diagnostic and prognostic markers, as well as for the development of more effective therapeutic strategies. Tumor invasion and metastasis involve interaction with and degradation of different components of the extracellular matrix (ECM). These processes are mediated by cell–ECM adhesion molecules and proteolytic enzymes, such as matrix metalloproteinases (MMPs) [5].

MMPs are zinc (Zn^2+^)-dependent endopeptidases, existing in two forms: intracellular and membrane-bound [6]. MMPs degrade ECM proteins such as fibronectin (FN), collagen, and laminin, and they play a key role in ECM remodeling in various physiological processes, like wound healing, tissue remodeling, organ morphogenesis, and angiogenesis. Under physiological conditions, MMPs are tightly regulated. Dysregulation of these enzymes is related to various diseases and pathological processes, like neurodegenerative, cardiovascular and lung diseases, arthritis, and tumor invasion [7]. Matrix metalloproteinases (MMPs) are tightly regulated at multiple levels, including gene transcription, pro-enzyme activation, and inhibition by endogenous tissue inhibitors of metalloproteinases (TIMPs), ensuring precise control over their proteolytic activity [8]. An imbalance between MMPs activation and inhibition plays a crucial role in the pathophysiology of cancer. In human cancers, MMPs are frequently overexpressed and have been implicated in nearly every stage of tumor development and progression. Their elevated levels and functional roles make them promising candidates as diagnostic and prognostic biomarkers across various cancer types [9,10]. Specifically, MMPs facilitate cancer cell proliferation, angiogenesis, and, crucially, enable tumor cells to escape the primary site and colonize distant organs by degrading the basement membrane and interstitial matrix components. This direct involvement in invasion and metastasis highlights MMPs as critical targets for cytostatic interventions aimed at preventing disease dissemination [11]. MMPs have been the object on several investigations in PTC, mostly restricted to MMP-9, demonstrating a higher expression, and it is associated with poor prognosis [6]. By contrast, other MMPs, including MMP-14, remain largely unexplored in the context of thyroid cancer.

We analyzed the gene expression data from PTC and normal thyroid tissue, obtained from The Cancer Genome Atlas (TCGA) Research Network, and found increased expression of *MMP-2*, *MMP-9*, and *MMP-14* in PTC samples. MMP-2 and MMP-9, classified as gelatinases, possess three FN type II-like repeats that enable them to effectively degrade gelatin and a broad range of extracellular matrix (ECM) components, including collagen, vitronectin, proteoglycans, and laminin. This degradative capability is essential to promote metastasis, as it facilitates the breakdown of the physical barrier of the ECM, thereby enabling tumor cells to invade surrounding tissues and disseminate to distant sites. [12]. MMP-14 activates proMMP-2 on the cell surface. It forms a trimolecular complex with tissue inhibitor of metalloproteinases 2 (TIMP-2) and proMMP-2, facilitating the conversion of proMMP-2 into its active form [13]. Recent studies have demonstrated that MMP-14 catalyzes pericellular collagen lysis, promoting tissue invasion of tumor cells [14].

To better elucidate the role of these MMPs, we conducted experiments on PTC cell lines that evidenced their role in migration and invasion. Our findings indicate that elevated MMP activity is a hallmark of aggressive PTC, suggesting that targeting MMPs, particularly with agents like NSC405020 and gallic acid, represents a promising therapeutic strategy to disrupt PTC tumor progression.

## 2. Results

### 2.1. MMPs Expression in Normal Thyroid Tissue and in PTC

**Figure 1:**
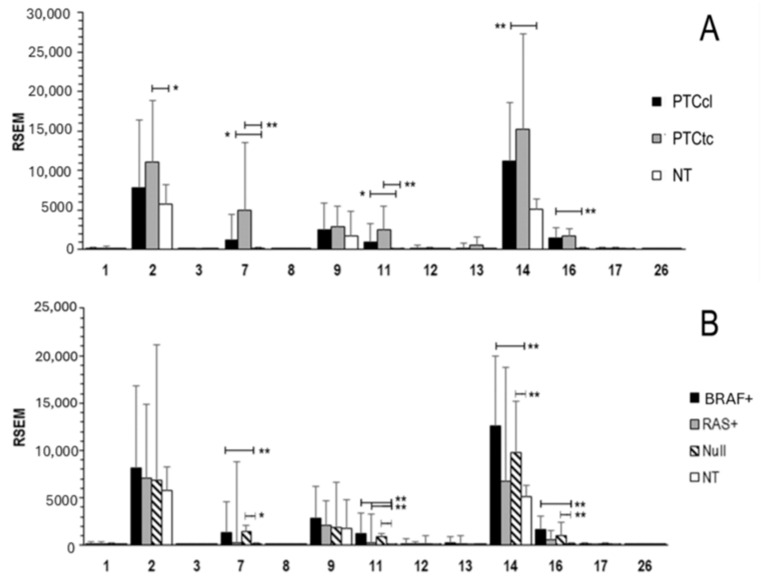
*MMPs’* expression in PTC and NT tissues: (**A**) mRNA expression of *MMPs* in PTCcl, PTCtc, and NT; and (**B**) mRNA expression of *MMPs* in PTC cases with *BRAFV600E* (*BRAF*^+^, N = 204), *RAS* mutation (*RAS*^+^, N = 19), or neither mutation (NULL, N = 132). Data are presented as mean ± standard deviation. Wilcoxon test: * *p* < 0.01, and ** *p* < 0.0001; RSEM, RNA-Seq by Expectation Maximization. PTCcl, classic type; PTCtc, tall cell.

We analyzed RNA-seq data from the TCGA-THCA dataset, comprising 355 papillary thyroid carcinoma (PTC) samples, including classical (PTCcl) and tall-cell (PTCtc) variants, and 58 normal thyroid (NT) tissue samples (Figure 1A). The results provide a comprehensive overview of the expression pattern of *MMP* gene family across the PTCcl, PTCtc, and NT. Among the matrix metalloproteinases present in the TCGA-THCA dataset, *MMP-2***,** *MMP-9***,** and *MMP-14* were the only members significantly expressed in normal thyroid (NT) samples. In both PTC subtypes, *MMP-7*, *MMP-11*, *MMP-14*, and *MMP-16* were consistently overexpressed, while *MMP-2* showed specific overexpression in the PTCtc subtype. The analysis was further refined by examining *MMPs’* expression in relation to driver gene mutations (Figure 1B). Notably, *RAS*-positive PTC displayed an expression profile similar to that of normal tissue (NT), with only *MMP-11* showing a modest yet significant overexpression.

### 2.2. Association of MMPs Expression and Clinical Features

We analyzed the correlation of MMPs with disease status and risk of tumor recurrence according to the 2015 American Thyroid Association guidelines [15] (Table 1). MMP-14 expression was positively correlated with all indicators of advanced disease, including lymph node metastasis (*p* = 0.0001), advanced stage (*p* = 0.0419), extrathyroidal extension (*p* = 0.0045), and increased risk of tumor recurrence (*p* = 0.0002). MMP16 expression was positively correlated with lymph node metastasis (*p* = 0.0012) and increased risk of tumor recurrence (*p* = 0.0003).

### 2.3. MMP-14 Expression and Activity in PTC Cell Lines

**Figure 2:**
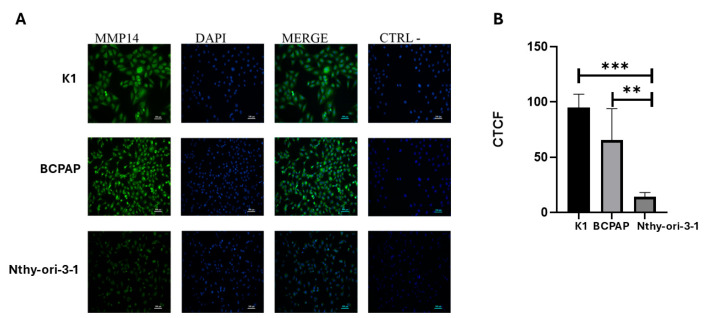
Immunofluorescence analysis of MMP-14 expression in thyroid cell lines. (**A**) Representative immunofluorescence images showing MMP-14 (green), DAPI-stained nuclei (blue), and merged channels (green and blue) in thyroid cell lines. A negative control (CTRL-) for MMP-14 staining is also shown. Scale bars represent 50 μm. (**B**) Quantitative analysis of MMP-14 expression, presented as Corrected Total Cell Fluorescence (CTCF). ** *p* = 0.0015; *** *p* < 0.0001. (Images corresponding to the data see Supplementary Materials).

**Figure 3:**
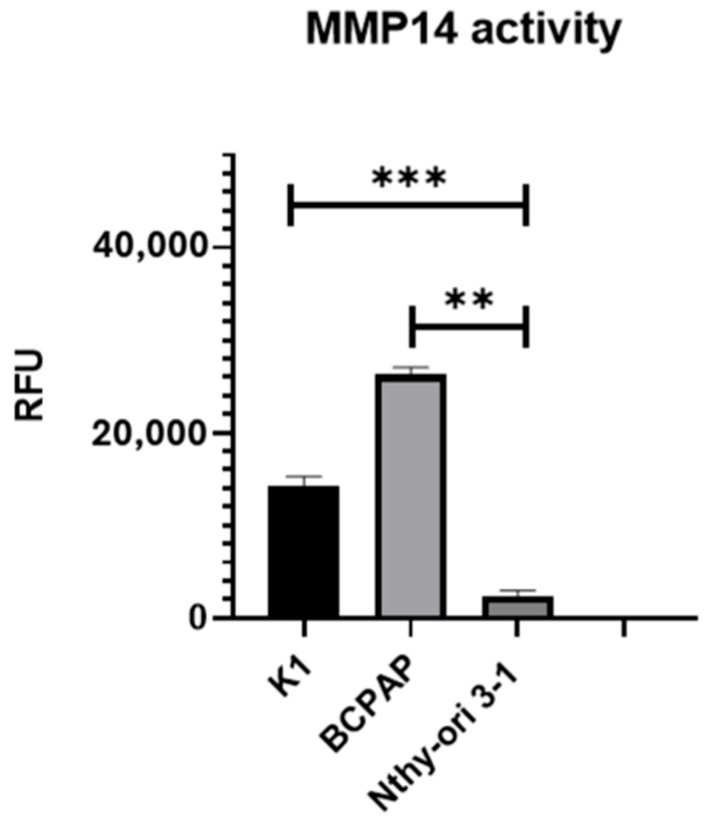
Enzymatic activity of MMP-14 in thyroid cell lines. Quantitative analysis of MMP-14 activity in K1 and BCPAP (PTC cell lines) compared to Nthy-ori 3-1 (non-tumoral immortalized thyroid cell line). MMP-14 activity is reported as mean relative fluorescence units (RFU) ± standard deviation. Statistical significance: ** *p* ≤ 0.01; *** *p* < 0.001.

Given the observed increase in MMP-14 expression in PTC tissues compared to normal thyroid tissue in the TCGA analysis, we sought to examine the expression levels of MMP-14 in a PTC cell-line model. MMP-14 protein expression was assessed by immunofluorescence with specific antibodies in the PTC cell lines K1 and BCPAP and compared to the immortalized non-tumoral thyroid cell line Nthy-ori 3-1 (Figure 2). K1 cells exhibited a 6.8-fold increase in MMP-14 expression compared to Nthy-ori 3-1 cells, while BCPAP displayed a 4.7-fold increase. We next investigated MMP-14 activity in these same cell lines (Figure 3). Consistent with the observed differential expression, marked differences in MMP-14 activities were also observed. However, in contrast to MMP-14 expression levels, its enzymatic activity was higher in BCPAP than in K1 cells. Specifically, BCPAP cells exhibited an 11-fold increase in activity compared to Nthy-ori 3-1, whereas K1 cells showed a 6-fold increase in activity. These results are consistent with the TCGA data and further support the evidence of elevated MMP-14 activity in thyroid cancer.

### 2.4. MMP-2 and MMP-9 Activity in PTC Cell Lines

**Figure 4:**
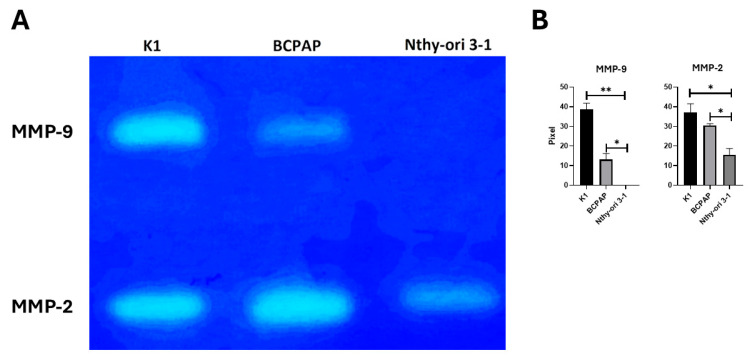
Specific gelatinase activity (MMP-2 and MMP-9) in thyroid cell lines by gelatin zymography. (**A**) Equal amounts of protein (30 µg) from cell culture-conditioned media were separated on a 7.5% SDS–polyacrylamide gel containing gelatin. Clear bands indicate the proteolytic activity of MMP-9 (upper band) and MMP-2 (lower band). (**B**) Densitometric analysis of the zymography bands was assessed using ImageJ software (NIH) version 1.54. Data are presented as mean ± SD. Statistical significance was determined by unpaired *t*-tests: * *p* < 0.05; ** *p* < 0.01.

MMP-14 activates proMMP-2 on the cell surface and, indirectly, MMP-9. To assess the activity of MMP-2 and MMP-9, we performed gelatin zymography (Figure 4). Gelatin zymography demonstrated a remarkable increase in MMP-9 activity in both PTC cell lines. Specifically, MMP-9 activity was minimal in Nthy-ori-3-1, while K1 cells exhibited a remarkable 386-fold increase (*p* = 0.003), and BCPAP cells showed a 131-fold increase (*p* = 0.027). The increase in MMP-2 activity in PTC cell lines was modest but significant, with K1 showing a 1.9-fold increase (*p* = 0.048), and BCPAP a 1.8-fold increase (*p* = 0.049). These results highlight the potential role of MMP-9 in this tumor.

### 2.5. Inhibition of MMP-14 Activity Reduces K1 Cell Migration and Invasion

**Figure 5:**
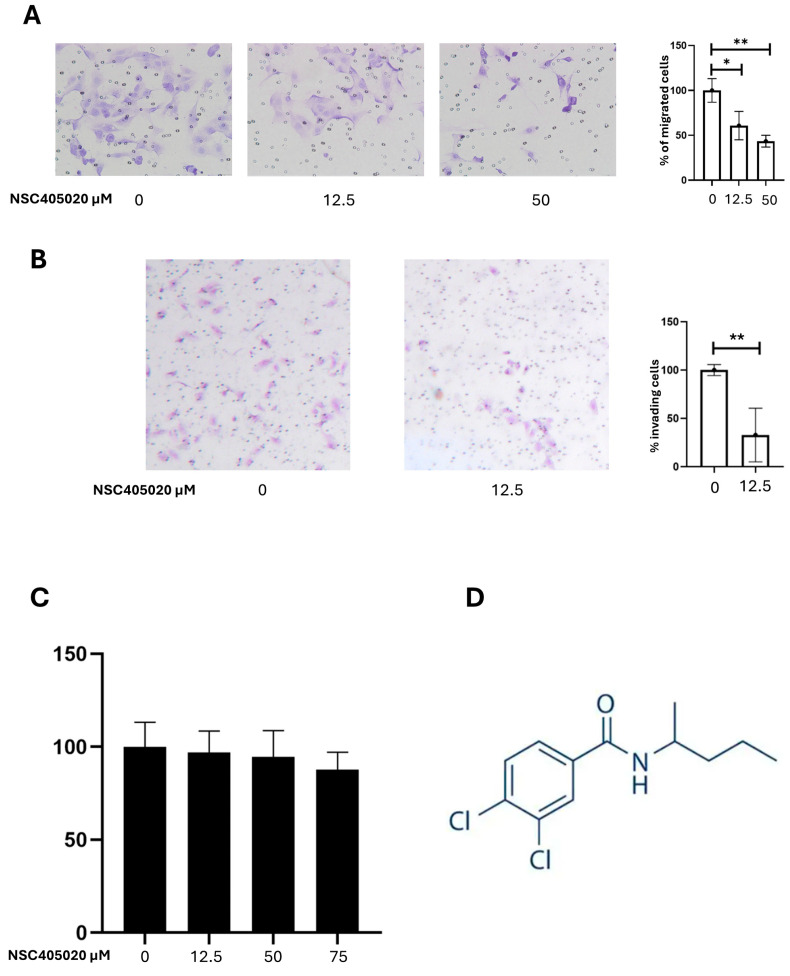
Effect of the MMP14 inhibitor NSC405020 on K1 cell migration, invasion, and viability. K1 cells were treated with the indicated concentrations of NSC405020 for 24 h and then subjected to (**A**) a transwell migration assay, using permeable supports, and (**B**) a Matrigel invasion assay. Data are presented as mean ± SEM from at least three independent experiments. Statistical significance was assessed using unpaired *t*-tests: * *p* < 0.05; ** *p* < 0.01. Representative images were captured at 20× magnification. (**C**) K1 cells were treated with NSC405020 or left untreated for 24 h. Cell viability was assessed by MTT assay and expressed as a percentage relative to untreated controls. No statistically significant differences in cell viability were observed. (**D**) Chemical structure of NSC405020.

To investigate the functional role of MMP-14 in PTC cell migration and invasion, we assessed the effect of NSC405020, an inhibitor of MMP-14 activity, on K1 cells, the cell line with more aggressive phenotype. Cell migration was evaluated using chamber migration assay, and cell invasion was assessed using Matrigel Invasion Assay. Treatment with NSC405020 significantly reduced the migratory capacity of K1 cells with a dose-dependent effect (Figure 5A). Treatment with 12.5 µM NSC405020 resulted in a 39.13% reduction in cell migration compared to the control (*p* = 0.033). Treatment with 50 µM NSC405020 led to an even more substantial 56.52% reduction in cell migration (*p* = 0.006). Similarly, NSC405020 treatment significantly impaired the invasive capacity of K1 cells in the Matrigel invasion assay (Figure 5B). Notably, treatment with 12.5 µM NSC405020 reduced the number of invasive cells by approximately 67.3% (*p* = 0.0055). The inhibitory effect of NSC405020 on cell migration and invasion was not due to cytotoxicity, as demonstrated by its minimal impact on cell viability at concentrations up to 100 µM in MTT assays (Figure 5C). Altogether, these results highlight MMP-14 as a mediator of cell migration and invasion.

### 2.6. Inhibition of MMP-2 and MMP-9 Activity Reduces K1 Cell Invasion and Migration

**Figure 6:**
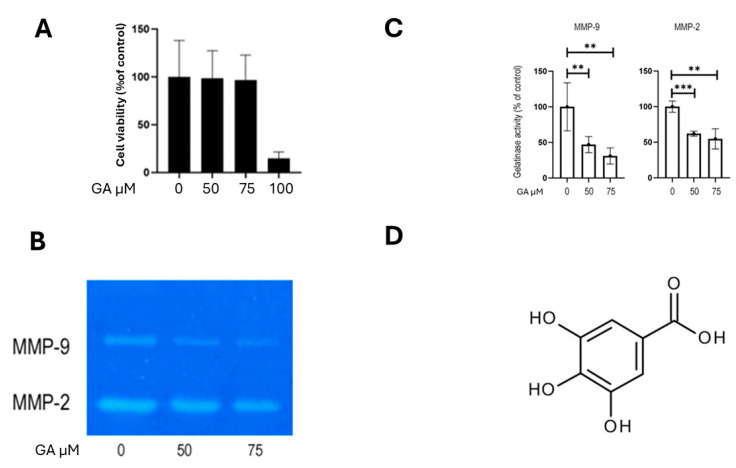
Effect of GA on MMP-2 and MMP-9 activity in K1 cells. (**A**) K1 cells were treated with increasing concentrations of gallic acid (GA) for 24 h, and cell viability was assessed by crystal violet staining. (**B**) Conditioned media were collected and analyzed by gelatin zymography to evaluate MMP2 and MMP9 enzymatic activity. (**C**) Densitometric quantification of zymographic bands relative to untreated controls. (**D**) Chemical structure of GA. Data are presented as mean ± SEM from at least three independent experiments. Statistical significance was assessed using unpaired *t*-tests: ** *p* < 0.005; *** *p* = 0.00008.

**Figure 7:**
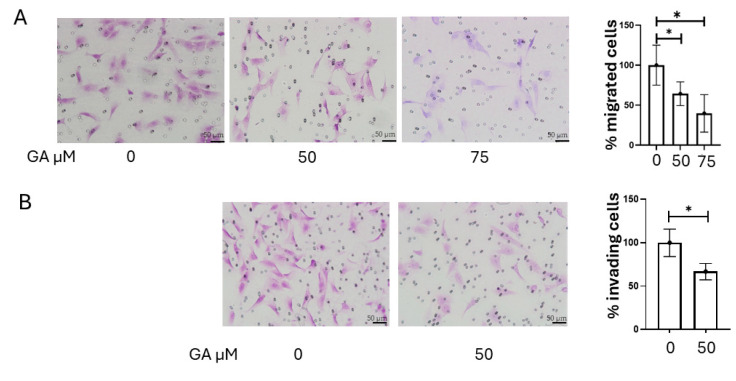
Effect of GA on K1 cell migration and invasion. K1 cells were treated with 50 µM or 75 µM GA for 24 h or left untreated and then seeded on permeable supports for cell migration assay (**A**) or used for Matrigel invasion assay (**B**). Cells that migrated to the lower side of the insert were fixed and stained with crystal violet. The number of migrated cells was counted and expressed as % of untreated cells. Data are presented as mean ± SEM of at least three independent experiments. Statistical significance was determined by unpaired *t*-tests: * *p* < 0.05.Representative images were captured at 20× magnification.

Inhibition of MMP-2 and MMP-9 activity was achieved with gallic acid (GA). GA-induced cytotoxicity was assessed using the crystal violet assay, as GA has been reported to interfere with formazan formation in MTT assays, potentially leading to inaccurate viability measurements [16]. The results indicated that GA exerted negligible cytotoxic effects on K1 cells at concentrations up to 75 µM (Figure 6A). We then evaluated the impact of gallic acid on MMP-2 and MMP-9 activity in K1 cells using gelatin zymography. Treatment with GA (0, 50, and 75 µM) resulted in a dose-dependent reduction in the activity of both MMP-2 and MMP-9 (Figure 6B). Compared to the control, MMP-9 activity was reduced by approximately 53% with 50 µM GA (*p* = 0.005), and by 69% with 75 µM GA (*p* = 0.002). Similarly, MMP-2 activity was reduced by approximately 38% in cells treated with 50 µM GA (*p* = 0.00008) and by 45% in cells treated with 75 µM GA (*p* = 0.001; Figure 6C). The chemical structure of GA is shown in Figure 6D. Then, we assessed the effect of GA on the invasive and migratory capabilities of K1 cells. Consistent with the observed reduction in MMP-2 and MMP-9 activity, GA treatment significantly reduced both the invasion and migration of K1 cells. To evaluate the effect of GA on cellular migration, a 24 h migration assay was performed on K1 cells. Treatment with 50 µM GA resulted in a 35.7% reduction in migration (*p* = 0.037), while 75 µM GA led to a 60.3% decrease in the number of migrated cells compared to the control (*p* = 0.022; Figure 7A). The 24 h invasion assay 50 µM GA caused a 33.3% reduction in invasive capacity (*p* = 0.032; Figure 7B). These findings highlight the contribution of MMP-2 and MMP-9 to the migratory and invasive behavior of PTC cells.

## 3. Discussion

In this study, we delved into the crucial role of MMPs, particularly MMP-14, MMP-2, and MMP-9, in PTC cell lines, and we explored the therapeutic potential of specific inhibitors, NSC405020 and GA, in modulating their activity, cell migration, and invasion. Several studies demonstrate that MMP-9 is overexpressed in PTC tissue and serum, correlating with aggressive clinicopathological features, such as lymph node metastasis, extrathyroidal invasion, and higher TNM stage [17,18,19,20]. Both total and active MMP-9 expression, especially when assessed by immunohistochemistry and zymography, are associated with poor prognosis and shorter disease-free survival [21,22]. The overall expression profile of MMPs in PTC has been far less explored, and no studies to date have focused on MMP-14.

Our findings consistently reinforce the established understanding of MMPs as pivotal mediators of ECM remodeling, a fundamental process in cancer progression, tumor invasion, and metastasis [23,24]. The tumor microenvironment, where MMPs exert their activity, is increasingly recognized as a vital therapeutic target in cancer [25,26]. This is particularly pertinent for PTC, where to understand the factors influencing prognosis, especially in aggressive or radioiodine-refractory cases, remains crucial for optimizing therapeutic strategies [27].

It is important to acknowledge that the intricate network of molecular events driving thyroid cancer progression also involves the prominent dysregulation of major signaling pathways, including the *MAPK* and *PI3K/AKT* pathways [28]. While our study specifically focuses on the therapeutic potential of MMP inhibition, understanding these broader molecular contexts is essential. The activities of MMPs can be influenced by cross-talk with these upstream oncogenic pathways, suggesting potential avenues for combined therapeutic strategies in the future to more comprehensively combat PTC aggressiveness.

The interaction between the ECM and its receptors, the integrins, has been extensively investigated in normal thyroid cell and in PTC, underscoring its role in thyroid cell physiology and promoting invasive behavior of PTC [29,30,31,32].

Our initial observations, derived from a meta-analysis of TCGA data, highlight a significant upregulation of some *MMPs* gene expression in PTC compared to normal thyroid tissue. Among these, *MMP-14* emerged as being particularly relevant, given its association with disease status and risk of tumor recurrence, as well as its well-documented ability to activate other *MMPs*, including *MMP-2* and *MMP-9*. This genomic insight guided our experimental approach. We subsequently confirmed this upregulation at the protein and activity level in a cell line model including two *BRAFV600E*-mutant PTC lines (K1 and BCPAP) and one non-tumoral thyroid cell line. Specifically, K1 cells exhibited a remarkable 6-fold increase in MMP-14 activity and a 6.8-fold increase in expression, while BCPAP cells showed an 11-fold increase in activity and a 4.7-fold increase in expression (Figure 2 and Figure 3). MMP-14, also known as MT1-MMP, is a promising drug target in various malignancies due to its direct involvement in tumor growth, migration, and collagen degradation [33]. The membrane-anchored nature of MT1-MMP, along with its distinct hemopexin (PEX) and catalytic domains, allows for unique inhibitory strategies that differ from broad-spectrum catalytic site inhibitors that have faced challenges in clinical trials [33,34,35].

Further investigation revealed an increase in gelatinase activity, primarily mediated by MMP-2 and MMP-9, in PTC cell lines. Gelatin zymography provided further validation, highlighting a pronounced elevation in MMP-9 activity in PTC cell lines, with a 386-fold increase in K1 and a 131-fold increase in BCPAP. These results align with the known mechanism by which MMP-14 activates latent gelatinases, particularly pro-MMP-2, a process crucial for extensive ECM degradation and tumor invasion [36,37,38]. The elevated activity of these type IV collagenases (MMP-2 and MMP-9) is recognized as a key contributor to tumor progression and dissemination in several malignancies, including osteosarcoma [39,40]. Indeed, the critical involvement of cell–matrix interactions, specifically integrin–fibronectin binding, further contributes to these invasive mechanisms in PTC [32]. This is particularly relevant in the context of PTC, where increased MMP-9 expression has been directly correlated with lymph node metastasis and poor prognosis [6,41,42].

To exploit the role of MMP-14 in cell migration and invasion and its potential as a target for therapy, we employed NSC405020, a selective inhibitor that targets the PEX domain of MMP-14, instead of its catalytic site. This distinctive feature is crucial, as broad-spectrum MMP inhibitors have often failed in clinical trials due to severe side effects as a consequence of non-specific inhibition of various MMPs involved in normal physiological processes [24]. NSC405020 acts by disrupting MMP-14 homodimerization, without having a direct effect on its catalytic activity, thus leading to increased collagen I deposition and thereby reducing tumor growth [33]. Further elucidating the mechanism, NSC405020 (3,4-Dichloro-N-(pentan-2-yl) benzamide) acts as a specific non-catalytic inhibitor of MMP-14. Its inhibitory action is mediated by a selective interaction with a particular binding pocket located within the PEX domain of MMP-14. This pocket is formed by key amino acid residues, including Met-328, Arg-330, Asp-376, Met-422, and Ser-470 of the enzyme. The specific structural features of NSC405020, notably the dichloro substituents on the benzamide group and the pentan-2-yl chain, are crucial for this selective recognition and binding to the PEX domain, which ultimately interferes with MMP-14 homodimerization without a direct effect on its catalytic activity [43]. Our results demonstrate that NSC405020 significantly reduced the migratory and invasive capacities of K1. Specifically, 50 µM of NSC405020 led to a 56.52% reduction in migration, and 12.5 µM of NSC405020 resulted in a 39.13% decrease in migration and a 67.3% reduction in invasion. These inhibitory effects were not attributable to cytotoxicity, further confirming MMP-14 as a critical factor in PTC cell motility and invasion, and supporting the concept of MMP-14 as a promising therapeutic target in malignancy [33]. The selective targeting of the unique PEX domain by NSC405020 offers a novel strategy for therapeutic intervention by selectively disabling MMP-14 regulatory function.

To further investigate the role of MMPs in the process of tumor invasion and dissemination, we investigated GA, a naturally occurring plant phenol with known anti-tumor properties, including the inhibition of MMP-2 and MMP-9 activity [16]. GA’s precise molecular mechanisms are multifaceted. The existing literature indicates that it primarily downregulates MMP-2 and MMP-9 expression and activity by modulating upstream signaling pathways. Specifically, GA has been shown to suppress key kinases, like *ERK*, *JNK*, *p38*, and *AKT*, and inhibit the *NF-κB* pathway, all critical for MMP regulation [16,44]. While some direct inhibition via zinc chelation is possible, GA’s main action appears to be through these broader modulations of intracellular signaling [45]. Our findings showed that GA significantly reduced the activity of both MMP-2 and MMP-9 in K1 cells in a dose-dependent manner (Figure 6B). Notable effects were observed in MMP-9 activity, with a reduction of 53% at 50 µM, and 69% at 75 µM, as well as in MMP-2 activity, with decreases of 38% at 50 µM, and 45% at 75 µM. Consistent with these enzymatic inhibitions, GA also significantly impaired the migratory (35.7% reduction at 50 µM; 60.3% at 75 µM) and invasive (33.3% reduction at 50 µM) capabilities of K1 cells, without exhibiting cytotoxicity (Figure 6A and Figure 7A,B). These results further support the existing literature on GA’s anticancer functions and its ability to modulate MMPs’ activity [16]. These results highlight GA’s potential as a therapeutic or adjuvant agent to inhibit pro-invasive and pro-migratory activities in PTC cells by targeting MMP-2 and MMP-9, offering a natural compound approach to complement existing therapies. While thyroid carcinoma typically has a favorable outcome, advanced and iodine-refractory cases necessitate specific treatment strategies based on molecularly targeted therapies or novel broad-spectrum agents [46,47,48].

## 4. Materials and Methods

### 4.1. Materials and Reagents

The following reagents were used in this study. Gallic acid and NSC405020 were both obtained from Sigma-Aldrich, St. Louis, MO, USA. For cell culture, we used DMEM/F12 and RPMI-1640 (Euroclone S.p.A, Pero, Italy), supplemented with Fetal Bovine Serum (FBS) (Gibco; Thermo Fisher Scientific, Inc., Waltham, MA, USA). Protein content was determined using the BCA method with reagents from Thermo Fisher Scientific, Inc., Waltham, MA, USA. The SensoLyte 520 MMP-14 assay kit (AnaSpec EGT Group, Fremont, CA, USA) was used for MMP-14 activity analysis. For gelatin zymography, gelatin (1 mg/mL; Cat. No. G2500) was purchased from Sigma-Aldrich, St. Louis, MO, USA. In immunofluorescence analyses, cells were fixed with an acetone–methanol 1:1 mixture. The MMP-14 polyclonal antibody (Cat. No. PA5-13183) was obtained from Invitrogen, Thermo Fisher Scientific, Inc. Waltham, MA, USA. VECTASHIELD Antifade Mounting Medium with DAPI (Vector Laboratories, Burlingame, CA, USA) was used for mounting slides. For cell-viability assays, 3-(4,5-dimethylthiazol-2-yl)-2,5-diphenyltetrazolium bromide (MTT) solution (4 mg/mL) and dimethyl sulfoxide (DMSO) were purchased from Sigma-Aldrich, St. Louis, MO, USA.

For cell-migration and -invasion assays, cell culture inserts (8 µm pore size) were obtained from Falcon, Corning (New York, NY, USA), and Corning Matrigel Invasion Chambers were obtained from Corning (New York, NY, USA). Crystal violet solution was used for cell staining (Sigma-Aldrich, St. Louis, MO, USA).

### 4.2. RNA-Seq Analysis

The TCGA-THCA dataset was retrieved from The Cancer Genome Atlas (TCGA) portal (https://portal.gdc.cancer.gov/ accessed on 2 January 2025). We analyzed raw-count gene expression data from 358 classical papillary thyroid carcinoma (PTCcl) samples, 37 tall cell-variant PTC (PTCtc) samples, and 58 normal thyroid (NT) solid tissue samples. Matching clinicopathological data were obtained from the cBioPortal for Cancer Genomics (https://www.cbioportal.org/ accessed on 2 January 2025) [49]. RNA-seq quantification was performed using the RNA-Seq by Expectation Maximization (RSEM) algorithm [50].

### 4.3. Cell Lines and Culture Mediums

BCPAP (Leibniz Institute DSMZ-GmbH, Braunschweig, Germany) and K1 (Merck KGaA, Darmstadt, Germany) are PTC cell lines harboring *BRAFV600E*. K1 cells were cultured in DMEM/F12 (Euroclone S.p.A, Pero, Italy), 10% FBS (Gibco; Thermo Fisher Scientific, Inc., USA). Nthy-ori-3-1 is a human thyroid follicular cell line derived from normal thyroid tissue, immortalized by transfection with a plasmid containing a defective genome of the SV40 virus (SV-ori), allowing them to retain several characteristics of normal thyroid follicular cells. BCPAP and Nthy-ori-3-1 were cultured in RPMI-1640 (Euroclone S.p.A, Pero, Italy), 10% FBS. Cells were cultured in a humidified incubator (5% CO_2_ and 95% air at 37 °C).

### 4.4. Fluorimetric MMP-14 Activity Assay

A total of 1.5 × 10^6^ cells were seeded in 100 mm culture plates and grown in the absence of serum. After 24 h, the cells were collected and washed 3 times with phosphate-buffered saline (PBS). Total proteins were extracted from the cells using the RIPA buffer method. The protein content of the cells was determined using the BCA method (Thermo Scientific, Waltham, MA, USA). MMP-14 activity was then measured using the SensoLyte 520 MMP-14 assay kit (AnaSpec EGT Group, Fremont, CA, USA) as follows: samples were incubated with 4-aminophenylmercuric acetate (APMA) for 3 h at 37 °C. Subsequently, 50 µL of each sample was combined with 50 µL of MMP-14 substrate solution in black 96-well microplates. The contents were gently mixed by shaking for 30 s and incubated at 37 °C for 60 min. Fluorescence intensity was measured at Ex/Em = 490 ± 20 nm/520 ± 20 nm. Once fluorescence readings are acquired, the data can be expressed in relative fluorescence units (RFU) after subtracting the background fluorescence from the substrate control well.

### 4.5. Immunofluorescence

Cells were plated onto sterile glass coverslips and cultured for 48 h at 37 °C in the specific medium and 10% FBS. Cells were rinsed in phosphate-buffered saline, pH 7.4 (PBS); fixed in acetone–methanol 1:1 (*v*/*v*) for 20 min; rinsed in PBS; and blocked with 1% BSA in PBS for 30 min. Cells were incubated with MMP-14 polyclonal antibody (PA5-13183 Invitrogen, Waltham, MA, USA) 1:100 in PBS and 0.2% Tween-20 overnight, washed in PBS, incubated with rabbit fluorescein-conjugated secondary antibody for 1 h, washed again, briefly rinsed in distilled water, mounted on microscope slides in Vectashield with DAPI (Vector Laboratories, Burligame, CA, USA), and then observed with a fluorescence microscope (Nikon Eclipse, Nikon Corporation, Tokyo, Japan).

### 4.6. Gelatin Gel Zymography

A total of 1.5 × 10^6^ cells were seeded in 100 mm tissue culture plates and grown in complete medium. Once cells reached 80% confluence, they were washed three times with sterile D-PBS, and fresh FBS-free medium was added. After 24 h of incubation, the conditioned media were collected and concentrated using Amicon^®^ Ultra Centrifugal Filter with a 30 kDa molecular weight cut-off (Amicon^®^ Ultra Centrifugal Filter, Merck KGaA, Darmstadt, Germany). Protein content was determined using the BCA method (Thermo Fisher Scientific, Inc., Waltham, MA, USA). Equal amounts of protein from conditioned media (30 µg) were analyzed by gelatin zymography under non-reducing conditions. Samples were loaded onto 7.5% SDS–polyacrylamide gel co-polymerized with gelatin 1 mg/mL (Cat. No. G2500, Sigma-Aldrich, St. Louis, MO, USA). Electrophoresis was performed at 100 V for 90–120 min at 4 °C. Following electrophoresis, gels were incubated for 1 h at room temperature in renaturation buffer containing 2.5% Triton X-100, 50 mM Tris-HCl pH 7.5, 5 mM CaCl_2_, and 1 µM ZnCl_2_. Gels were then incubated at 37 °C for 48 h in development buffer (50 mM Tris-HCl pH 7.5, 200 mM NaCl, 5 mM CaCl_2_, and 5 µM ZnCl_2_) to allow for substrate degradation. After incubation, gels were stained with 0.5% Coomassie Brilliant Blue R-250 and destained using a solution of 50% methanol and 5% acetic acid to visualize proteolytic bands.

### 4.7. Cell Migration and Matrigel Invasion Assays

K1 PTC cells were cultured in appropriate media until reaching 80–90% confluence. Cells were then trypsinized, and the cell number was determined using a hemocytometer. For the cell migration assay, cell culture inserts with an 8 µm pore size (Falcon, Corning, New York, NY, USA) were used, whereas for the cell Matrigel invasion assay, Corning Matrigel Invasion Chambers (Corning, New York, NY, USA) were used. Cells were treated with different concentrations of drugs for 24 h. In total, 25,000 treated and 25,000 untreated (control) cells were seeded in the upper chamber of the permeable supports in serum-free medium. The lower chamber of each well contained medium supplemented with 5% serum as a chemoattractant. The cells were incubated for 24 h at 37 °C in a humidified atmosphere containing 5% CO_2_. After the 24 h incubation period, non-migrated cells remaining in the upper chamber were carefully removed using a cotton-tipped swab. Cells that had migrated through the pores to the lower side of the insert were fixed with 4% paraformaldehyde and then stained with crystal violet. The number of migrated cells was quantified by counting the cells under an Olympus microscope at 4× magnification. Images were analyzed using ImageJ software (NIH) version 1.54 to determine the number of migrated cells per field.

### 4.8. Cell-Viability (MTT) Assay

Cell viability was assessed using the 3-(4,5-dimethylthiazol-2-yl)-2,5-diphenyltetrazolium bromide (MTT) assay. K1 PTC cells were seeded in 96-well plates at a density of 5 × 10^3^ cells/well and allowed to adhere overnight. Cells were then treated with varying concentrations of NSC405020 (0, 12.5, 50, and 75 µM; Sigma-Aldrich, St. Louis, MO, USA) for 24 h. Following incubation, 4 mg/mL of MTT solution (Sigma-Aldrich, St. Louis, MO, USA) was added to each well, and cells were incubated for an additional 4 h at 37 °C. The supernatant was then removed, and 100 µL of dimethyl sulfoxide (DMSO) was added to each well to dissolve the formazan crystals. Absorbance was measured at 490 nm using a microplate reader. All experiments were performed in triplicate.

### 4.9. Crystal Violet-Based Cell-Viability Assay

Cell viability was assessed via a modified crystal violet staining assay. Cells were treated with various concentrations of gallic acid for 24 h. Following treatment, the cells were washed with PBS and fixed with a fixative solution for 30 min. After washing with distilled water, cells were stained with a crystal violet solution for 30 min. Excess dye was removed by washing with distilled water. The number of adherent stained cells was directly counted under a microscope to quantify viable cells and determine the cytotoxic effects of gallic acid.

### 4.10. Statical Analysis

Data are presented as mean ± standard deviation (SD). The normality of distribution was assessed using the Shapiro–Wilk test. For normally distributed variables, comparisons were performed using the paired Student’s *t*-test, whereas non-normally distributed variables were analyzed using the Wilcoxon signed-rank test. Differences in integrin expression and clinical features among groups were evaluated using one-way analysis of variance (ANOVA), with statistical significance set at *p* < 0.01. Univariate regression analyses were conducted using the Spearman rank correlation test. All statistical analyses were performed using SPSS Statistics software, version 26 (IBM Corp., Armonk, NY, USA).

## 5. Conclusions

Our study provides robust evidence that elevated MMP activity is a hallmark of aggressive PTC, highlighting MMPs’ critical role in thyroid cancer progression. These findings suggest that targeting MMP-14 and its downstream effectors, MMP-2 and MMP-9, may represent a viable and promising therapeutic strategy for PTC. Future research should use in vivo models and clinical studies to further validate these therapeutic targets and rigorously assess their efficacy and safety within the complex biological context of a living organism.

## Figures and Tables

**Table 1:** Mean MMP gene expression and analysis of the correlation with nodal status, disease stage, extrathyroidal extension and risk group ^a^. Only significant data are reported.

Nodal Status						
**Gene**	**N0**		**N1**		***p***	
MMP14	9735		13,210		0.0001	
MMP16	1172		1657		0.0012	
**Disease stage**						
	**I**	**II**	**III**	**IV**		***p***
MMP14	10,454	10,313	12,475	13,769		0.0419
**Extra thyroidal extension**						
	**None**	**Minimal**	**Moderate/advanced**		***p***	
MMP14	10,439	13,385	14,231		0.0045	
**Risk group ^a^**						
	**Low**	**Intermediate**	**High**		***p***	
MMP14	9196	11,875	15,930		0.0002	
MMP16	979	1615	1797		0.0003	

^a^, estimated risk of tumor recurrence based on the 2015 American Thyroid Association guidelines. Statistical analysis was ANOVA for disease stage, extrathyroidal extension and risk group, and Wilcoxcon for nodal status. Mean gene expression is calculated by RSEM.

## Data Availability

The data presented in this study are openly available from The Cancer Genome Atlas (TCGA) repository, specifically the TCGA-THCA dataset, accessible via platforms such as cBioPortal for Cancer Genomics (https://www.cbioportal.org/ accessed on 2 January 2025).

## Supplementary Materials

The following supporting information can be downloaded at: https://www.mdpi.com/article/10.3390/ijms26167956/s1. The following supplementary figures are provided to ensure full data transparency and reproducibility. They include uncropped and unedited images of the experiments presented in the main manuscript. Figures S1.1–S1.12: These figures show the uncropped and unedited immunofluorescence images corresponding to the data presented in Figure 2 of the main text. Figure S2: This figure presents the uncropped and unedited gelatin zymography gel used to assess the gelatinase activity in the tumor cell lines K1 and BCPAP and the non-tumoral cell line Nthy-ori-3-1, as shown in Figure 4 of the main text. Figures S3.1–S3.3: These figures show the uncropped and unedited images of the migration assay for K1 cells treated with 0, 12.5, and 50 µM of NSC405020. This data corresponds to Figure 5A in the main text. Figures S4.1–S4.2: These figures present the uncropped and unedited images of the invasion assay after K1 cell treatment with 0 and 12.5 µM of NSC405020. This data corresponds to Figure 5B in the main text. Figure S5: This figure shows the uncropped and unedited gelatin zymography gel for K1 cells treated with 0, 50, and 75 µM of Gallic Acid, as seen in Figure 6B of the main text. Figures S6.1–S6.3: These figures represent the uncropped and unedited images from the migration assay of K1 cells treated with 0, 50, and 75 µM of GA. This data corresponds to Figure 7A of the main text. Figures S7.1–S7.2: These figures present the uncropped and unedited images from the invasion assay for K1 cells treated with 0 and 50 µM of GA. This data corresponds to Figure 7B of the main text.

## Abbreviations

The following abbreviations are used in this manuscript:

ANOVA	analysis of variance
APMA	4-aminophenylmercuric acetate
BCA	bicinchoninic acid
BSA	bovine serum albumin
CO_2_	carbon dioxide
CTCF	Corrected Total Cell Fluorescence
DMEM/F12	Dulbecco’s Modified Eagle Medium/Nutrient Mixture F-12
DMSO	dimethyl sulfoxide
ECM	extracellular matrix
FBS	Fetal Bovine Serum
FN	fibronectin
MMP	matrix metalloproteinase
MTT	3-(4,5-dimethylthiazol-2-yl)-2,5-diphenyltetrazolium bromide
NT	normal thyroid
PBS	phosphate-buffered saline
PEX	hemopexin domain
PTC	papillary thyroid carcinoma
PTCcl	classical papillary thyroid carcinoma
PTCtc	tall cell-variant papillary thyroid carcinoma
RFU	relative fluorescence units
RPMI-1640	Roswell Park Memorial Institute 1640 medium
RSEM	RNA-Seq by Expectation Maximization
SD	standard deviation
TCGA	The Cancer Genome Atlas
TIMP	tissue inhibitor of metalloproteinases

## References

[1] Lloyd R.V., Erickson L.A., Casey M.B., Lam K.Y., Lohse C.M., Asa S.L., Chan J.K.C., DeLellis R.A., Harach H.R., Kakudo K. (2004). Observer Variation in the Diagnosis of Follicular Variant of Papillary Thyroid Carcinoma. Am. J. Surg. Pathol..

[2] Limaiem F., Rehman A., Mazzoni T. (2025). Papillary Thyroid Carcinoma. StatPearls.

[3] Sun J.-H., Li Y.-R., Chang K.-H., Liou M.-J., Lin S.-F., Tsai S.-S., Yu M.-C., Hsueh C., Chen S.-T. (2022). Evaluation of Recurrence Risk in Patients with Papillary Thyroid Cancer through Tumor-Node-Metastasis Staging: A Single-Center Observational Study in Taiwan. Biomed. J..

[4] Nabhan F., Dedhia P.H., Ringel M.D. (2021). Thyroid Cancer, Recent Advances in Diagnosis and Therapy. Int. J. Cancer.

[5] Curran S., Murray G.I. (1999). Matrix Metalloproteinases in Tumour Invasion and Metastasis. J. Pathol..

[6] Li Z., Wei J., Chen B., Wang Y., Yang S., Wu K., Meng X. (2023). The Role of MMP-9 and MMP-9 Inhibition in Different Types of Thyroid Carcinoma. Molecules.

[7] Scannevin R.H., Alexander R., Haarlander T.M., Burke S.L., Singer M., Huo C., Zhang Y.-M., Maguire D., Spurlino J., Deckman I. (2017). Discovery of a Highly Selective Chemical Inhibitor of Matrix Metalloproteinase-9 (MMP-9) That Allosterically Inhibits Zymogen Activation. J. Biol. Chem..

[8] Hadler-Olsen E., Fadnes B., Sylte I., Uhlin-Hansen L., Winberg J. (2011). Regulation of Matrix Metalloproteinase Activity in Health and Disease. FEBS J..

[9] Egeblad M., Werb Z. (2002). New Functions for the Matrix Metalloproteinases in Cancer Progression. Nat. Rev. Cancer.

[10] Huang H. (2018). Matrix Metalloproteinase-9 (MMP-9) as a Cancer Biomarker and MMP-9 Biosensors: Recent Advances. Sensors.

[11] Kessenbrock K., Plaks V., Werb Z. (2010). Matrix Metalloproteinases: Regulators of the Tumor Microenvironment. Cell.

[12] Fanjul-Fernández M., Folgueras A.R., Cabrera S., López-Otín C. (2010). Matrix Metalloproteinases: Evolution, Gene Regulation and Functional Analysis in Mouse Models. Biochim. Biophys. Acta Mol. Cell Res..

[13] Nagase H., Visse R., Murphy G. (2006). Structure and Function of Matrix Metalloproteinases and TIMPs. Cardiovasc. Res..

[14] Adley B.P., Gleason K.J., Yang X.J., Stack M.S. (2009). Expression of Membrane Type 1 Matrix Metalloproteinase (MMP-14) in Epithelial Ovarian Cancer: High Level Expression in Clear Cell Carcinoma. Gynecol. Oncol..

[15] Haugen B.R., Alexander E.K., Bible K.C., Doherty G.M., Mandel S.J., Nikiforov Y.E., Pacini F., Randolph G.W., Sawka A.M., Schlumberger M. (2016). 2015 American Thyroid Association Management Guidelines for Adult Patients with Thyroid Nodules and Differentiated Thyroid Cancer: The American Thyroid Association Guidelines Task Force on Thyroid Nodules and Differentiated Thyroid Cancer. Thyroid.

[16] Liu K.-C., Huang A.-C., Wu P.-P., Lin H.-Y., Chueh F.-S., Yang J.-S., Lu C.-C., Chiang J.-H., Meng M., Chung J.-G. (2011). Gallic Acid Suppresses the Migration and Invasion of PC-3 Human Prostate Cancer Cells via Inhibition of Matrix Metalloproteinase-2 and -9 Signaling Pathways. Oncol. Rep..

[17] Maeta H., Ohgi S., Terada T. (2001). Protein Expression of Matrix Metalloproteinases 2 and 9 and Tissue Inhibitors of Metalloproteinase 1 and 2 in Papillary Thyroid Carcinomas. Virchows Arch..

[18] Marečko I., Cvejić D., Šelemetjev S., Paskaš S., Tatić S., Paunović I., Savin S. (2014). Enhanced Activation of Matrix Metalloproteinase-9 Correlates with the Degree of Papillary Thyroid Carcinoma Infiltration. Croat. Med. J..

[19] Zarkesh M., Zadeh-Vakili A., Akbarzadeh M., Fanaei S.A., Hedayati M., Azizi F. (2018). The Role of Matrix Metalloproteinase-9 as a Prognostic Biomarker in Papillary Thyroid Cancer. BMC Cancer.

[20] Ivković I., Limani Z., Jakovčević A., Huić D., Prgomet D. (2022). Role of Matrix Metalloproteinases and Their Inhibitors in Locally Invasive Papillary Thyroid Cancer. Biomedicines.

[21] Šelemetjev S., Đorić I., Paunović I., Tatić S., Cvejić D. (2016). Coexpressed High Levels of VEGF-C and Active MMP-9 Are Associated with Lymphatic Spreading and Local Invasiveness of Papillary Thyroid Carcinoma. Am. J. Clin. Pathol..

[22] Xu D., Su C., Guo L., Yan H., Wang S., Yuan C., Chen G., Pang L., Zhang N. (2019). Predictive Significance of Serum MMP-9 in Papillary Thyroid Carcinoma. Open Life Sci..

[23] Coussens L.M., Werb Z. (1996). Matrix Metalloproteinases and the Development of Cancer. Chem. Biol..

[24] Overall C.M., Kleifeld O. (2006). Tumour Microenvironment—Opinion: Validating Matrix Metalloproteinases as Drug Targets and Anti-Targets for Cancer Therapy. Nat. Rev. Cancer.

[25] Fidler I.J. (2002). The Organ Microenvironment and Cancer Metastasis. Differentiation.

[26] Xiao T., Takagi J., Coller B.S., Wang J.-H., Springer T.A. (2004). Structural Basis for Allostery in Integrins and Binding to Fibrinogen-Mimetic Therapeutics. Nature.

[27] Marotta V., Rocco D., Crocco A., Deiana M.G., Martinelli R., Di Gennaro F., Valeriani M., Valvano L., Caleo A., Pezzullo L. (2024). Survival Predictors of Radioiodine-Refractory Differentiated Thyroid Cancer Treated with Lenvatinib in Real Life. J. Clin. Endocrinol. Metab..

[28] Prete A., Borges De Souza P., Censi S., Muzza M., Nucci N., Sponziello M. (2020). Update on Fundamental Mechanisms of Thyroid Cancer. Front. Endocrinol..

[29] Vitale M., Illario M., Di Matola T., Casamassima A., Fenzi G., Rossi G. (1997). Integrin Binding to Immobilized Collagen and Fibronectin Stimulates the Proliferation of Human Thyroid Cells in Culture. Endocrinology.

[30] Vitale M. (1998). Fibronectin Is Required to Prevent Thyroid Cell Apoptosis through an Integrin-Mediated Adhesion Mechanism. J. Clin. Endocrinol. Metab..

[31] Mautone L., Ferravante C., Tortora A., Tarallo R., Giurato G., Weisz A., Vitale M. (2021). Higher Integrin Alpha 3 Beta1 Expression in Papillary Thyroid Cancer Is Associated with Worst Outcome. Cancers.

[32] Rocco D., Tortora A., Marotta V., Machado A.M., Selistre-de-Araujo H.S., Vitale M. (2025). Integrin-Fibronectin Interaction Is a Pivotal Biological and Clinical Determinant in Papillary Thyroid Carcinoma. Endocr. Relat. Cancer.

[33] Remacle A.G., Golubkov V.S., Shiryaev S.A., Dahl R., Stebbins J.L., Chernov A.V., Cheltsov A.V., Pellecchia M., Strongin A.Y. (2012). Novel MT1-MMP Small-Molecule Inhibitors Based on Insights into Hemopexin Domain Function in Tumor Growth. Cancer Res..

[34] Nagase H., Woessner J.F. (1999). Matrix Metalloproteinases. J. Biol. Chem..

[35] Chakraborti S., Mandal M., Das S., Mandal A., Chakraborti T. (2003). Regulation of Matrix Metalloproteinases: An Overview. Mol. Cell Biochem..

[36] Sato H., Takino T., Okada Y., Cao J., Shinagawa A., Yamamoto E., Seiki M. (1994). A Matrix Metalloproteinase Expressed on the Surface of Invasive Tumour Cells. Nature.

[37] Strongin A.Y., Collier I., Bannikov G., Marmer B.L., Grant G.A., Goldberg G.I. (1995). Mechanism of Cell Surface Activation of 72-kDa Type IV Collagenase. Isolation of the Activated Form of the Membrane Metalloprotease. J. Biol. Chem..

[38] Okada A., Tomasetto C., Lutz Y., Bellocq J.P., Rio M.C., Basset P. (1997). Expression of Matrix Metalloproteinases during Rat Skin Wound Healing: Evidence That Membrane Type-1 Matrix Metalloproteinase Is a Stromal Activator of pro-Gelatinase A. J. Cell Biol..

[39] Zhang L., Shi J., Feng J., Klocker H., Lee C., Zhang J. (2004). Type IV Collagenase (Matrix Metalloproteinase-2 and -9) in Prostate Cancer. Prostate Cancer Prostatic Dis..

[40] Bjørnland K., Flatmark K., Pettersen S., Aaasen A.O., Fodstad O., Maelandsmo G.M. (2005). Matrix Metalloproteinases Participate in Osteosarcoma Invasion. J. Surg. Res..

[41] Guan H., Guo Y., Liu L., Ye R., Liang W., Li H., Xiao H., Li Y. (2018). INAVA Promotes Aggressiveness of Papillary Thyroid Cancer by Upregulating MMP9 Expression. Cell Biosci..

[42] Zhang B.-T., Li Y., Jiang Q.-L., Jiang R., Zeng Y., Jiang J. (2024). Human Adipose-Derived Stem Cells Promote Migration of Papillary Thyroid Cancer Cell via Leptin Pathway. Ann. Med..

[43] Vieira D., Barralet J., Harvey E.J., Merle G. (2022). Detecting the PEX Like Domain of Matrix Metalloproteinase-14 (MMP-14) with Therapeutic Conjugated CNTs. Biosensors.

[44] Kuo C.-L., Lai K.-C., Ma Y.-S., Weng S.-W., Lin J.-P., Chung J.-G. (2014). Gallic Acid Inhibits Migration and Invasion of SCC-4 Human Oral Cancer Cells through Actions of NF-κB, Ras and Matrix Metalloproteinase-2 and -9. Oncol. Rep..

[45] Pang J.-H.S., Yen J.-H., Wu H.-T., Huang S.-T. (2017). Gallic Acid Inhibited Matrix Invasion and AP-1/ETS-1-Mediated MMP-1 Transcription in Human Nasopharyngeal Carcinoma Cells. Int. J. Mol. Sci..

[46] Libertini S., Iacuzzo I., Ferraro A., Vitale M., Bifulco M., Fusco A., Portella G. (2007). Lovastatin Enhances the Replication of the Oncolytic Adenovirus Dl1520 and Its Antineoplastic Activity against Anaplastic Thyroid Carcinoma Cells. Endocrinology.

[47] Giuffrida D., Prestifilippo A., Scarfia A., Martino D., Marchisotta S. (2012). New Treatment in Advanced Thyroid Cancer. J. Oncol..

[48] Cherifi F., Awada A. (2025). Molecular Oncology of Iodine Refractory Thyroid Cancer Current Therapies and Perspective. Crit. Rev. Oncol./Hematol..

[49] Gao J., Aksoy B.A., Dogrusoz U., Dresdner G., Gross B., Sumer S.O., Sun Y., Jacobsen A., Sinha R., Larsson E. (2013). Integrative Analysis of Complex Cancer Genomics and Clinical Profiles Using the cBioPortal. Sci. Signal..

[50] Li B., Dewey C.N. (2011). RSEM: Accurate Transcript Quantification from RNA-Seq Data with or without a Reference Genome. BMC Bioinform..

