# Supplementary figures and images for: Inhibition of Metalloproteinases-2, -9, and -14 Suppresses Papillary Thyroid Carcinoma Cell Migration and Invasion
Source: Int J Mol Sci. 2025 Aug 18;26(16):7956. doi: 10.3390/ijms26167956 (PMC12386556; doi:10.3390/ijms26167956)

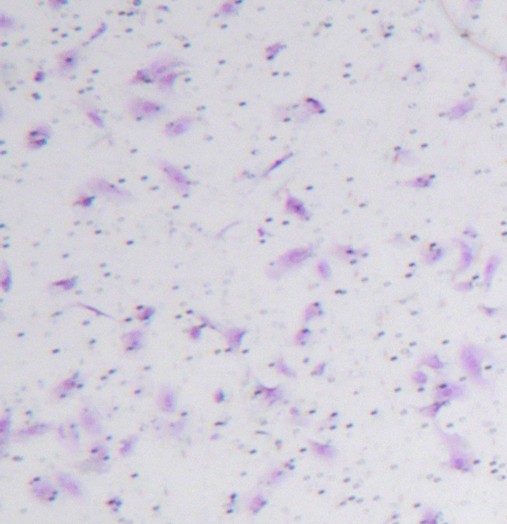

Supplement: Supplementary file 1 [file ijms-26-07956-s001.zip › NSC405020/invasion assay/Figure 5B K1 0uM nsc405020.jpg]

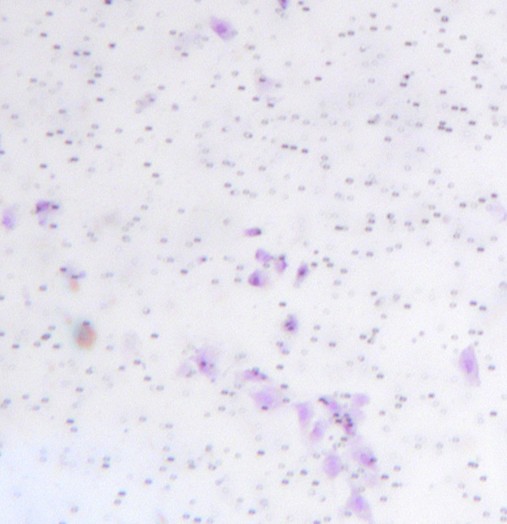

Supplement: Supplementary file 1 [file ijms-26-07956-s001.zip › NSC405020/invasion assay/Figure 5B K1 12.5uM nsc405020.jpg]

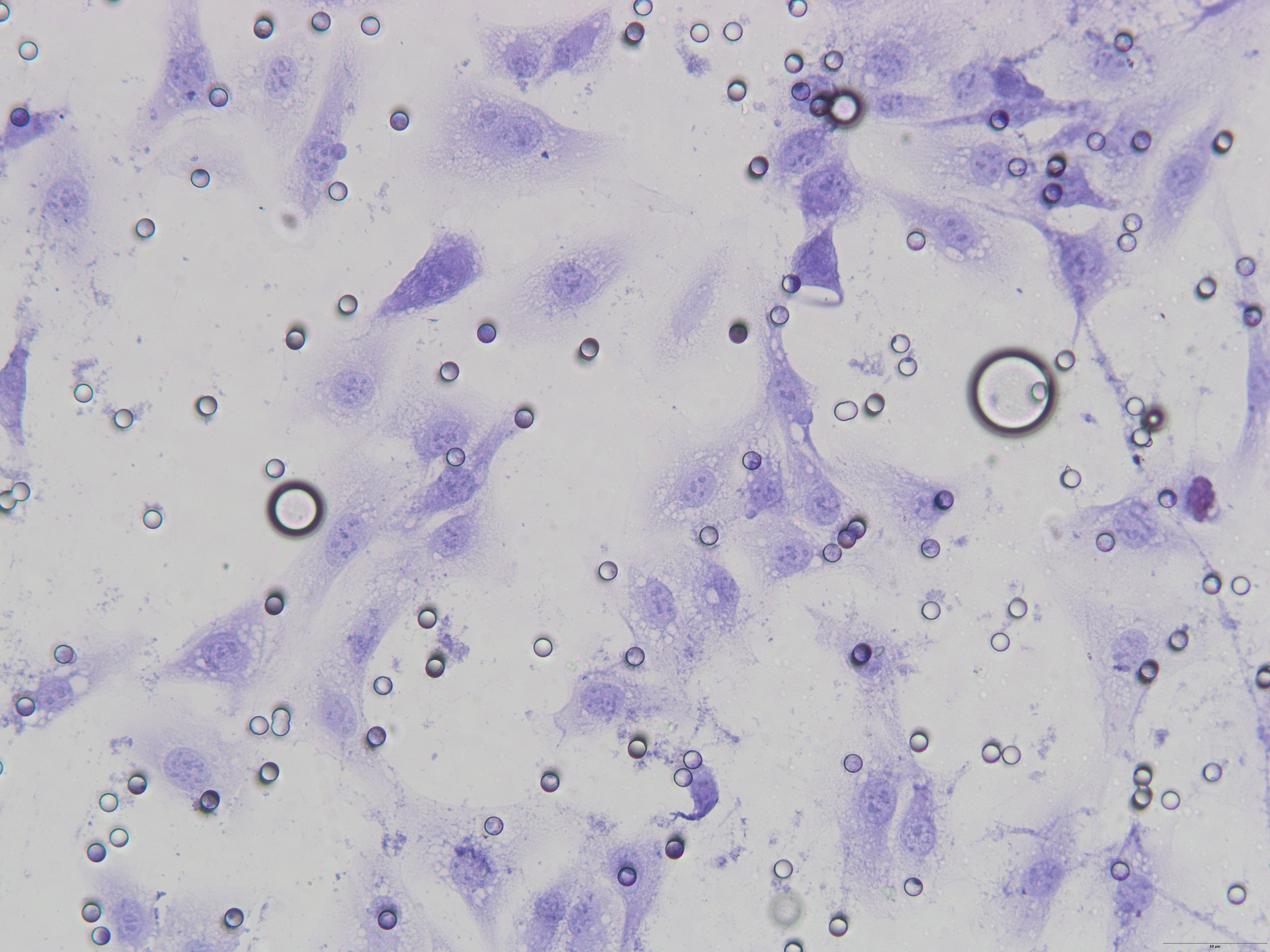

Supplement: Supplementary file 1 [file ijms-26-07956-s001.zip › NSC405020/migration assay/Figure 5 A K1 nsc405020 0 uM 20x.jpg]

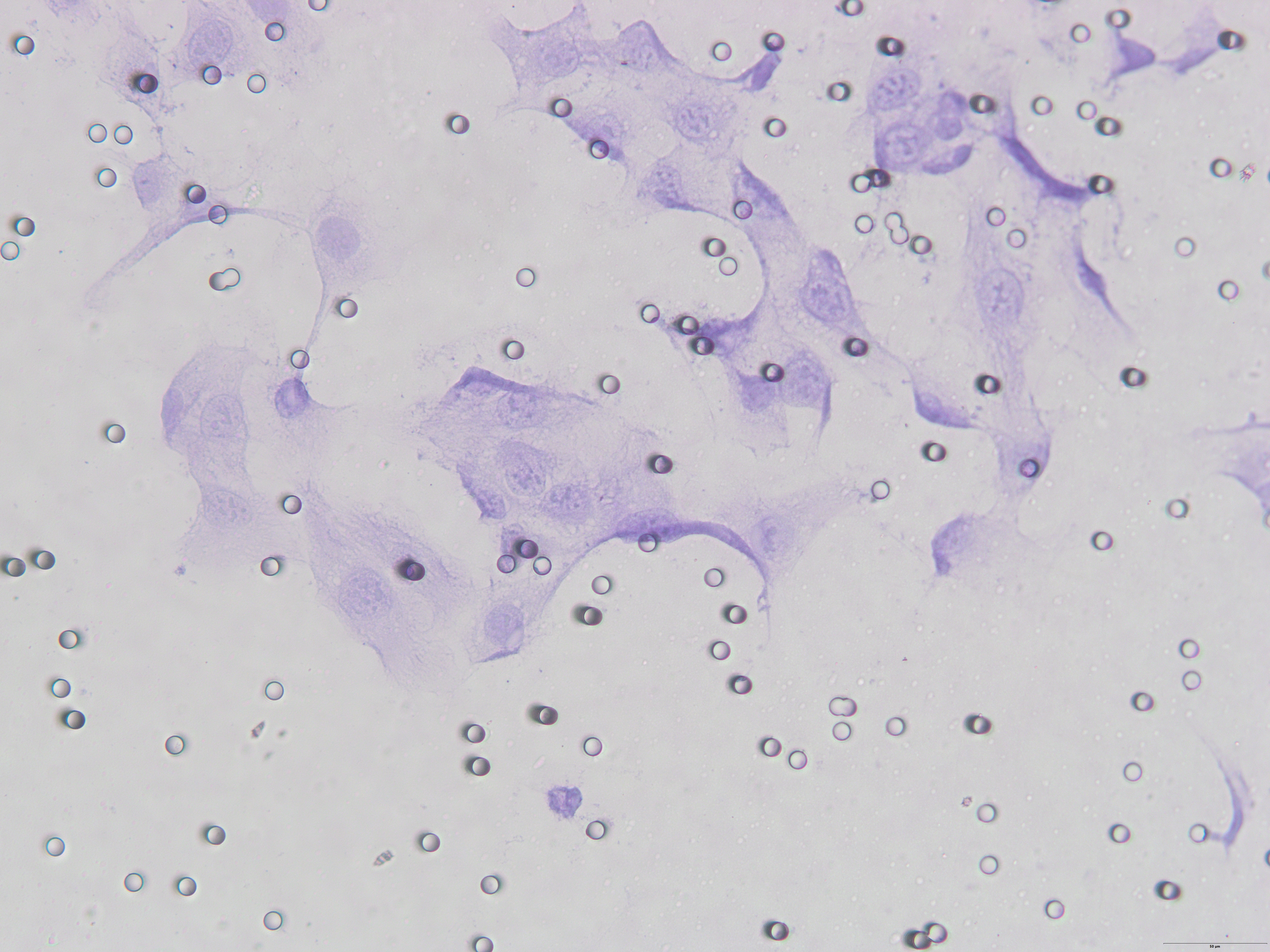

Supplement: Supplementary file 1 [file ijms-26-07956-s001.zip › NSC405020/migration assay/Figure 5 A K1 nsc405020 12.5uM 20x.jpg]

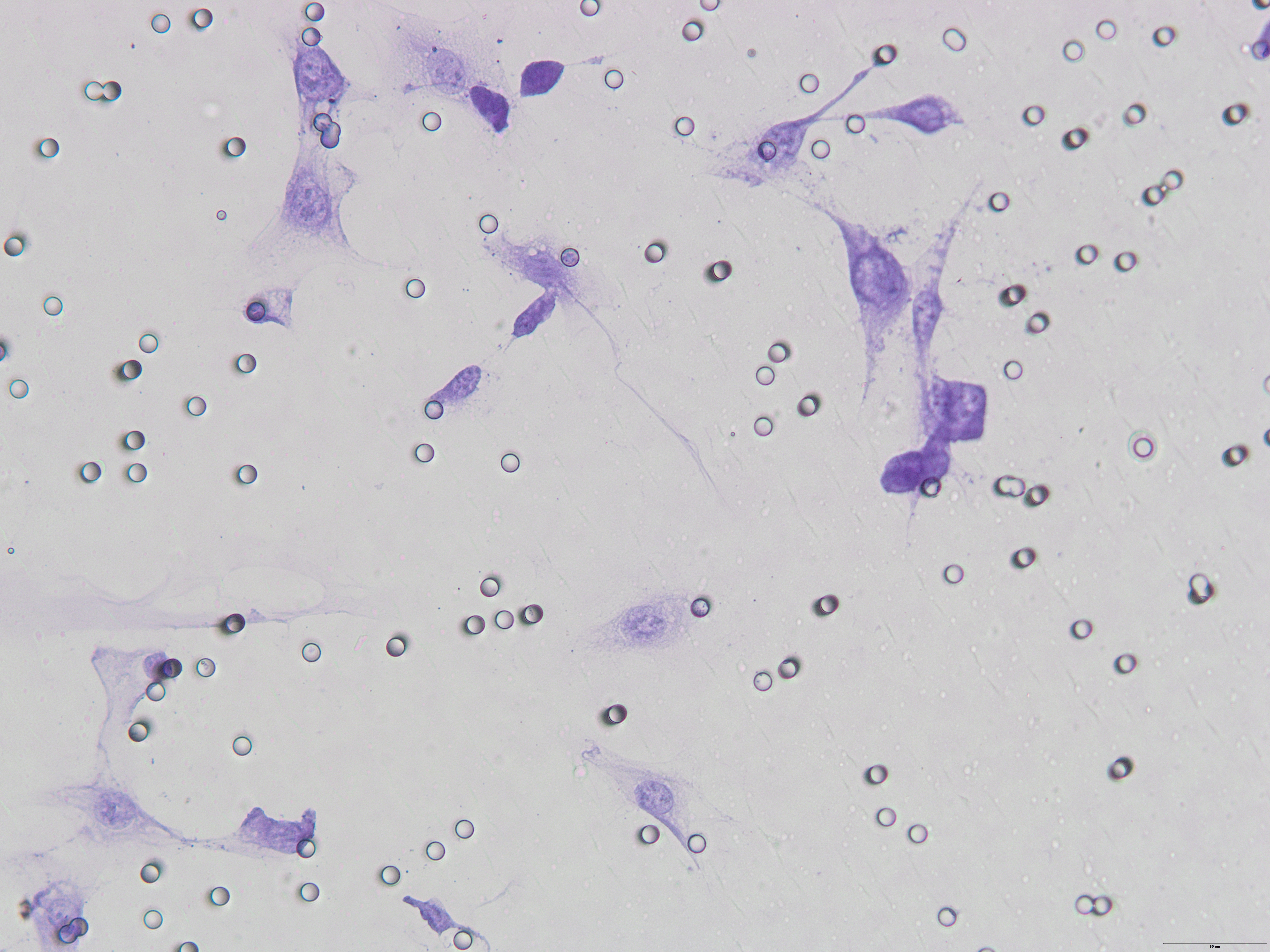

Supplement: Supplementary file 1 [file ijms-26-07956-s001.zip › NSC405020/migration assay/Figure 5 A K1 nsc405020 50uM 20x.jpg]

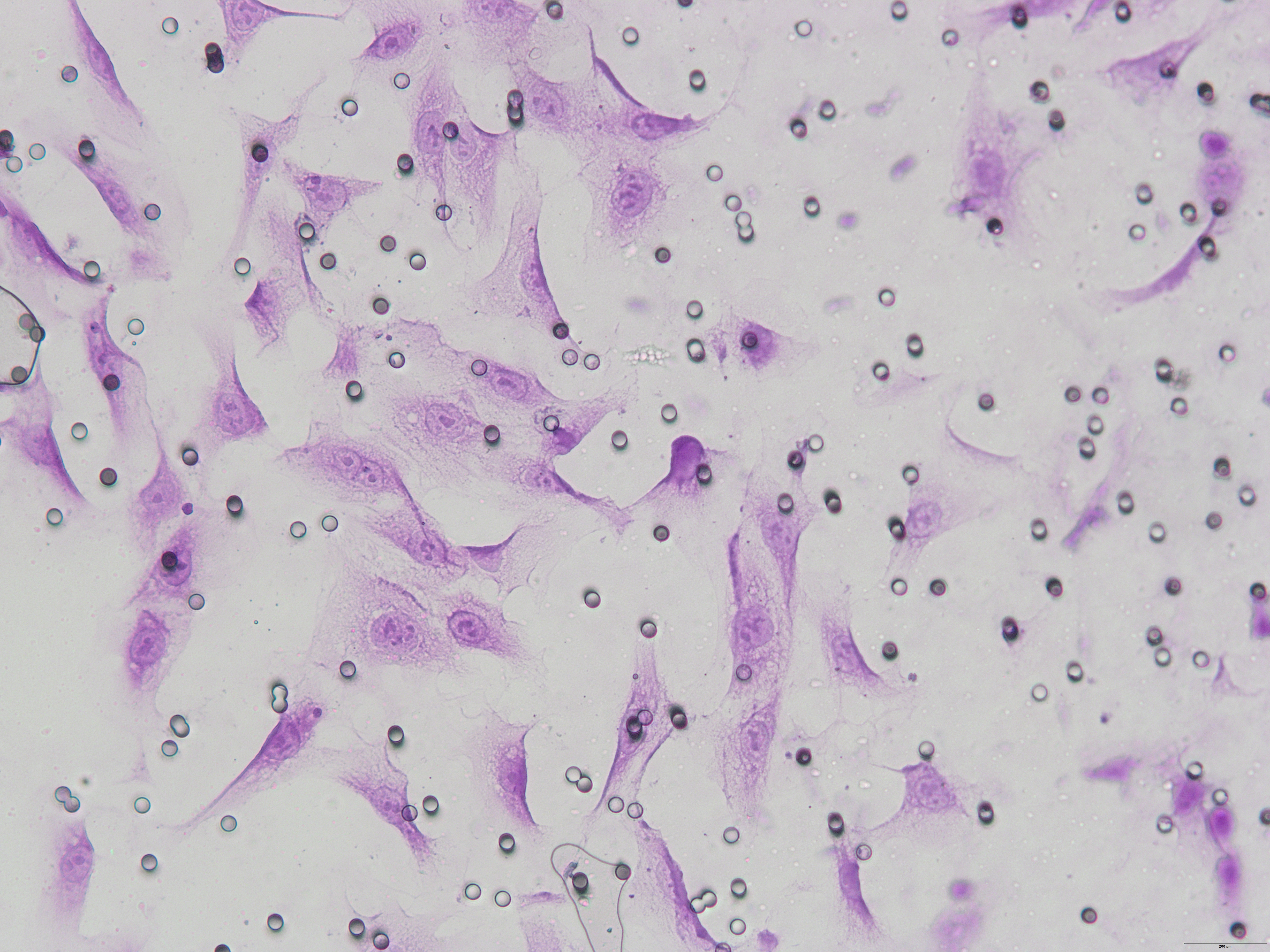

Supplement: Supplementary file 1 [file ijms-26-07956-s001.zip › AG/Invasion assay/Figure 7 B k1 0 uM AG 20X.jpg]

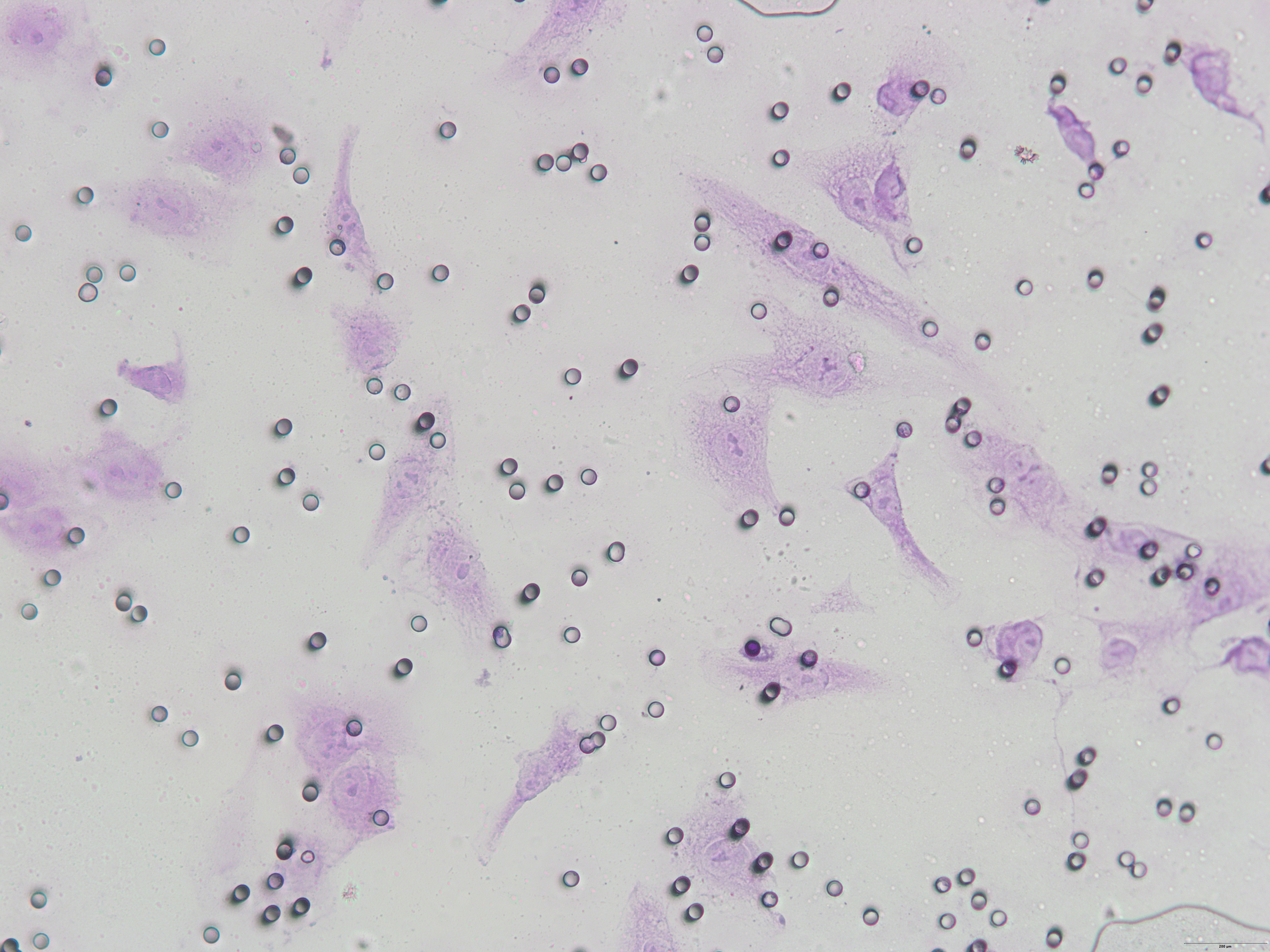

Supplement: Supplementary file 1 [file ijms-26-07956-s001.zip › AG/Invasion assay/Figure 7 B k1 50 uM AG 20X.jpg]

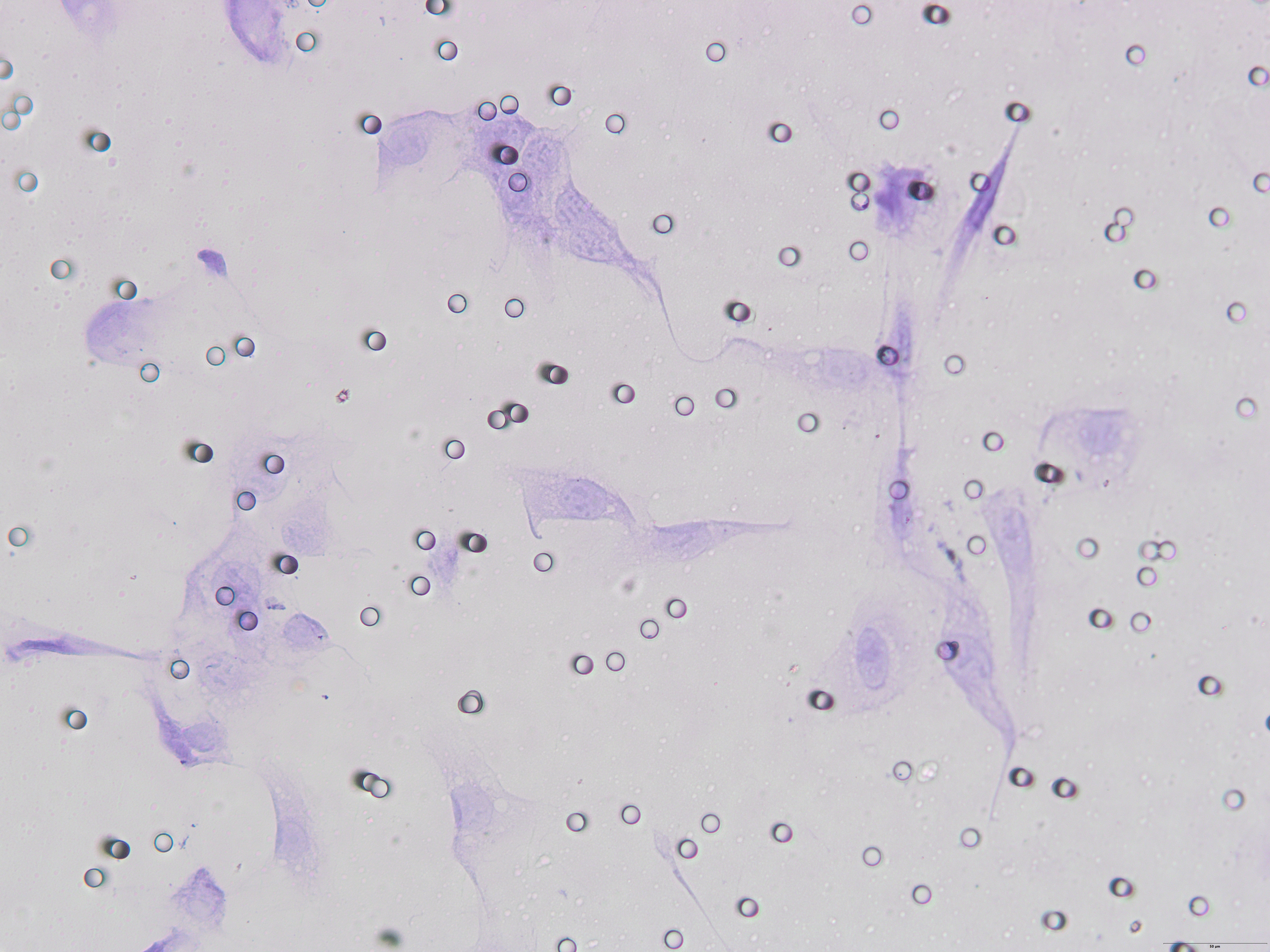

Supplement: Supplementary file 1 [file ijms-26-07956-s001.zip › AG/Migration assay/Figure 7 A 75 uM AG 20X.jpg]

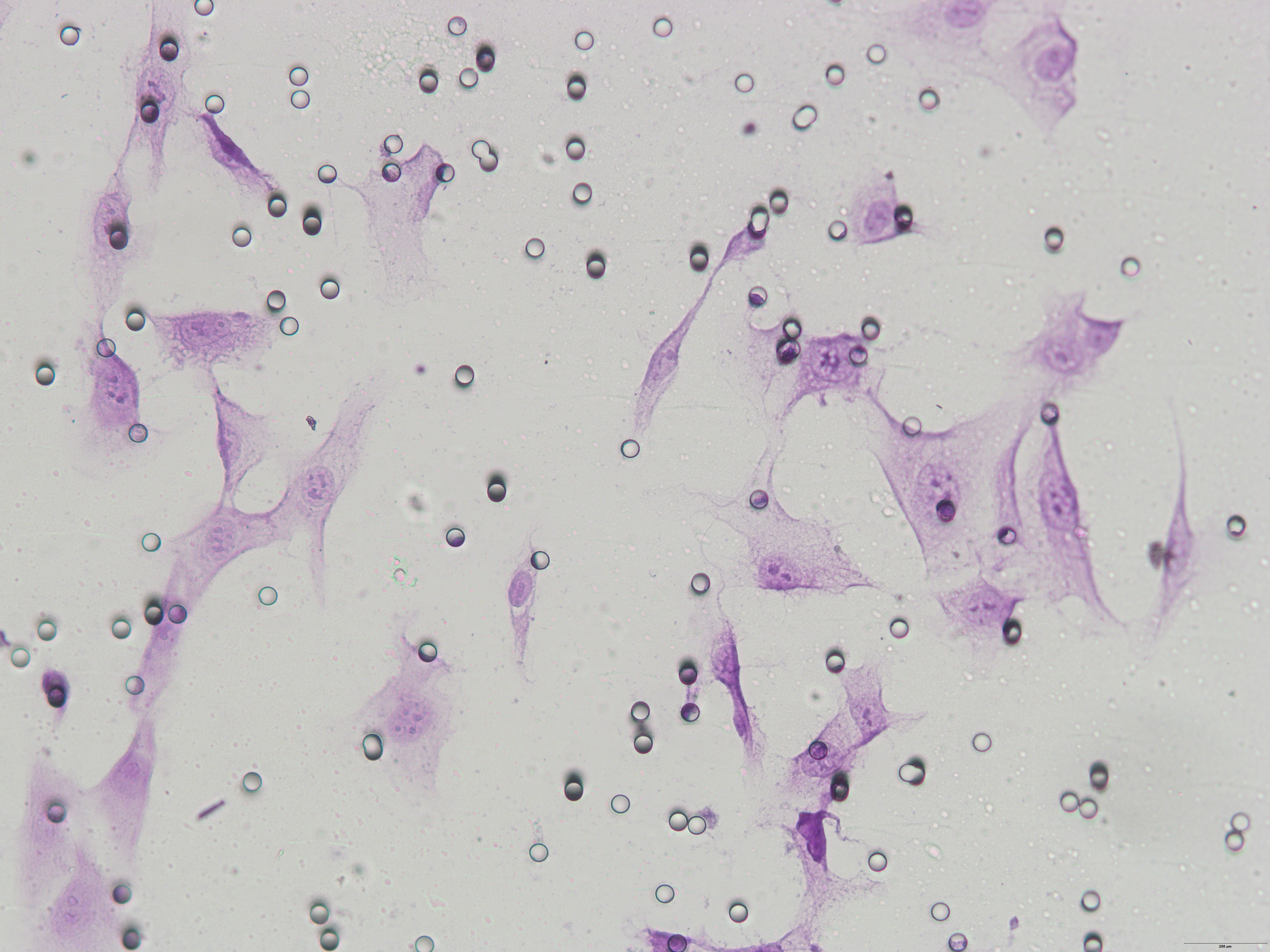

Supplement: Supplementary file 1 [file ijms-26-07956-s001.zip › AG/Migration assay/Figure 7 A k1 50 uM AG 20X.jpg]

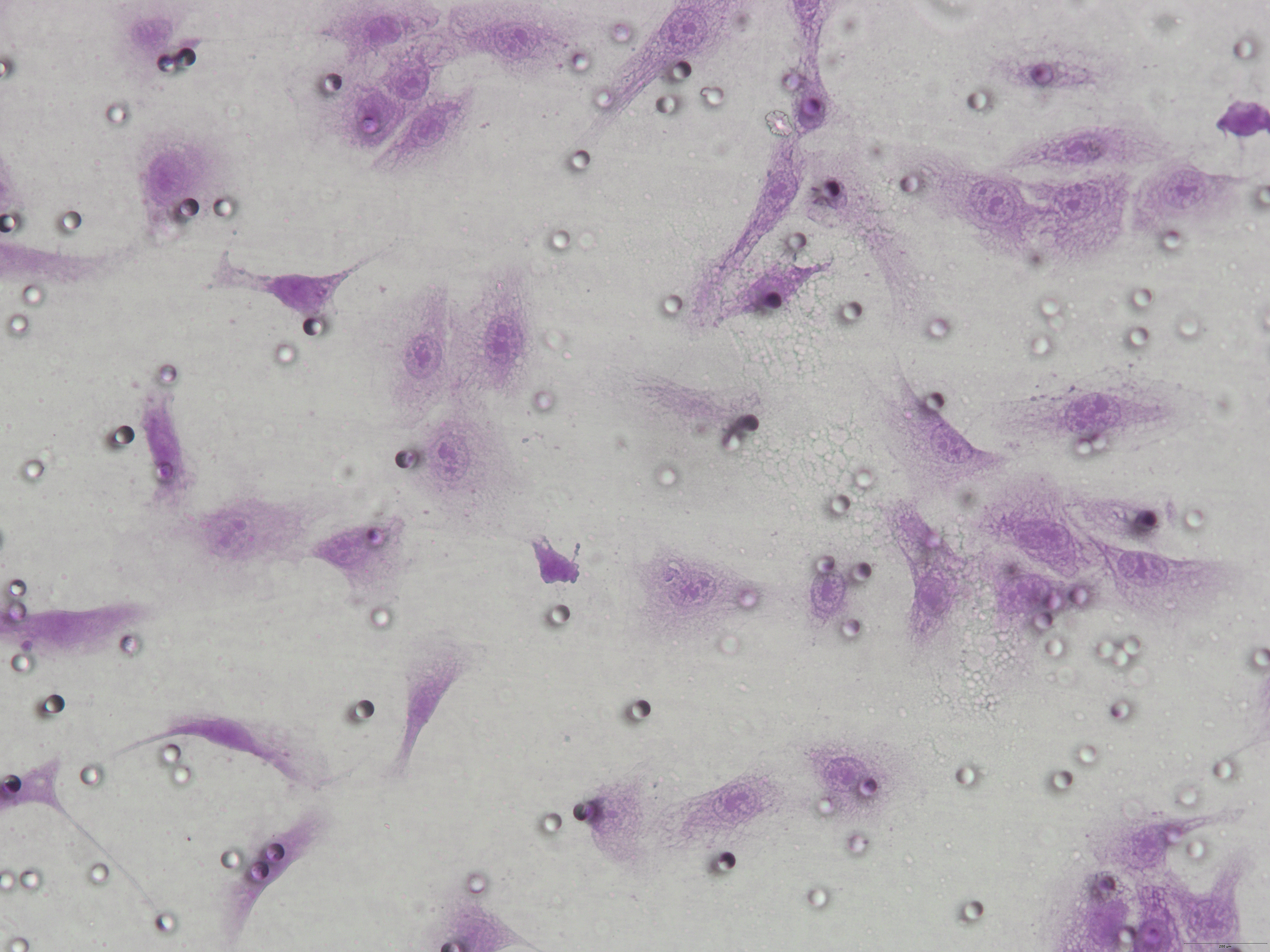

Supplement: Supplementary file 1 [file ijms-26-07956-s001.zip › AG/Migration assay/Figure 7 A k1 0 uM 20X.jpg]

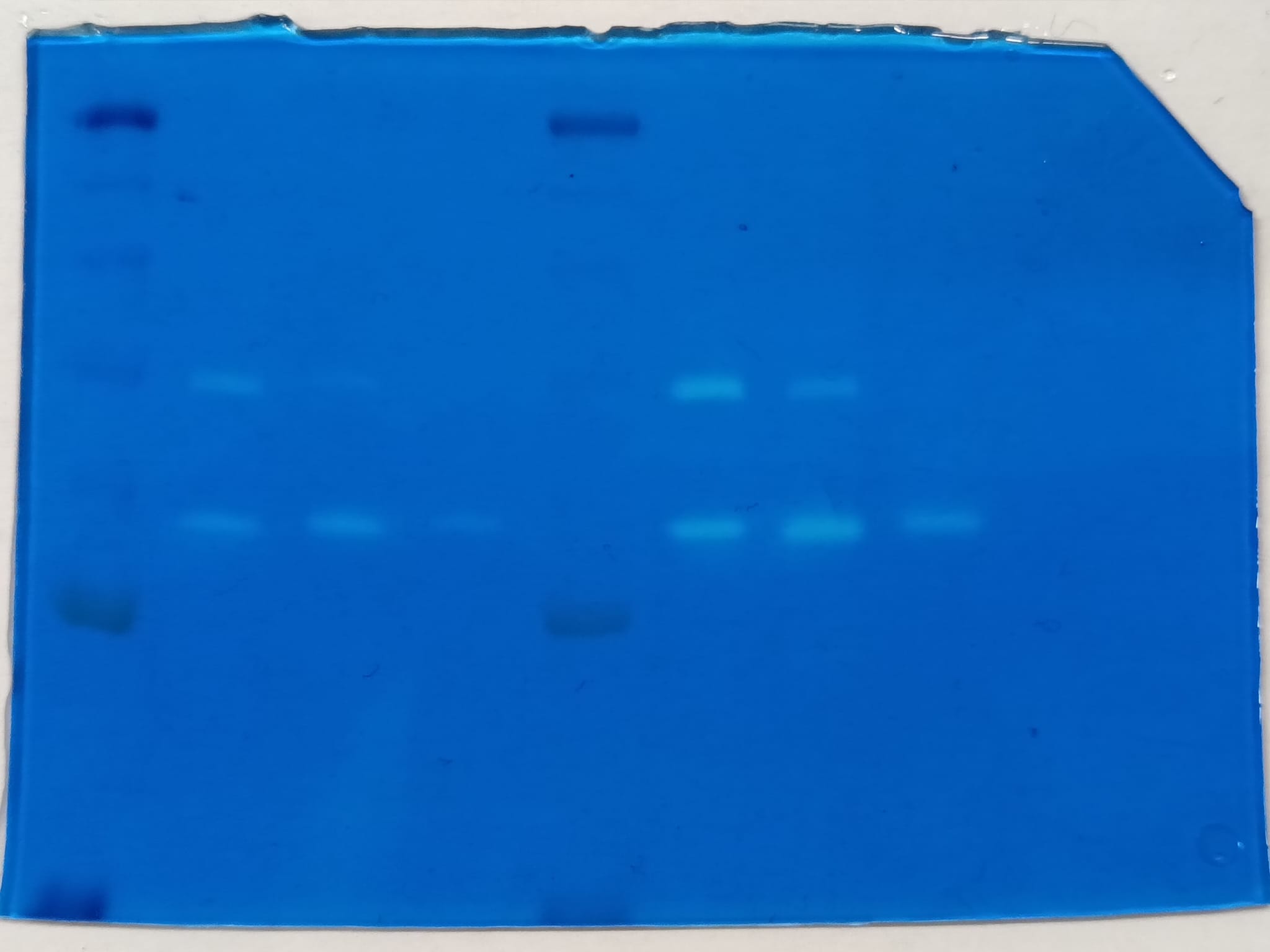

Supplement: Supplementary file 1 [file ijms-26-07956-s001.zip › gelatin zymography/Figure 4A.jpeg]

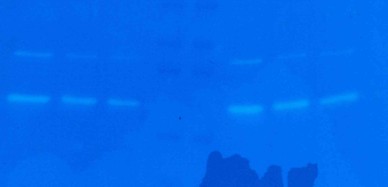

Supplement: Supplementary file 1 [file ijms-26-07956-s001.zip › gelatin zymography/Figure 6B.jpg]

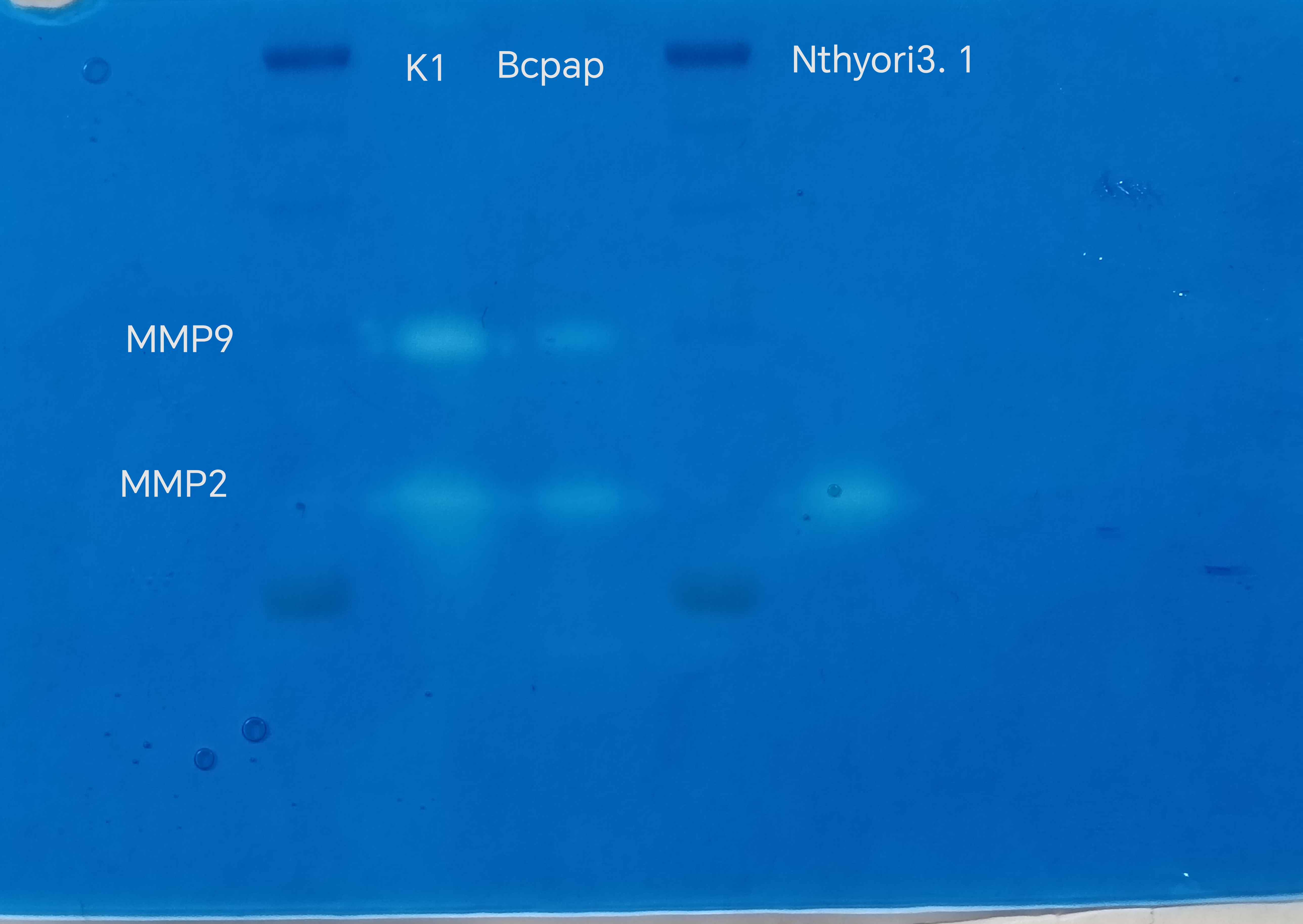

Supplement: Supplementary file 1 [file ijms-26-07956-s001.zip › gelatin zymography/zimografia k1 bcpap nthyori3.1.jpg]

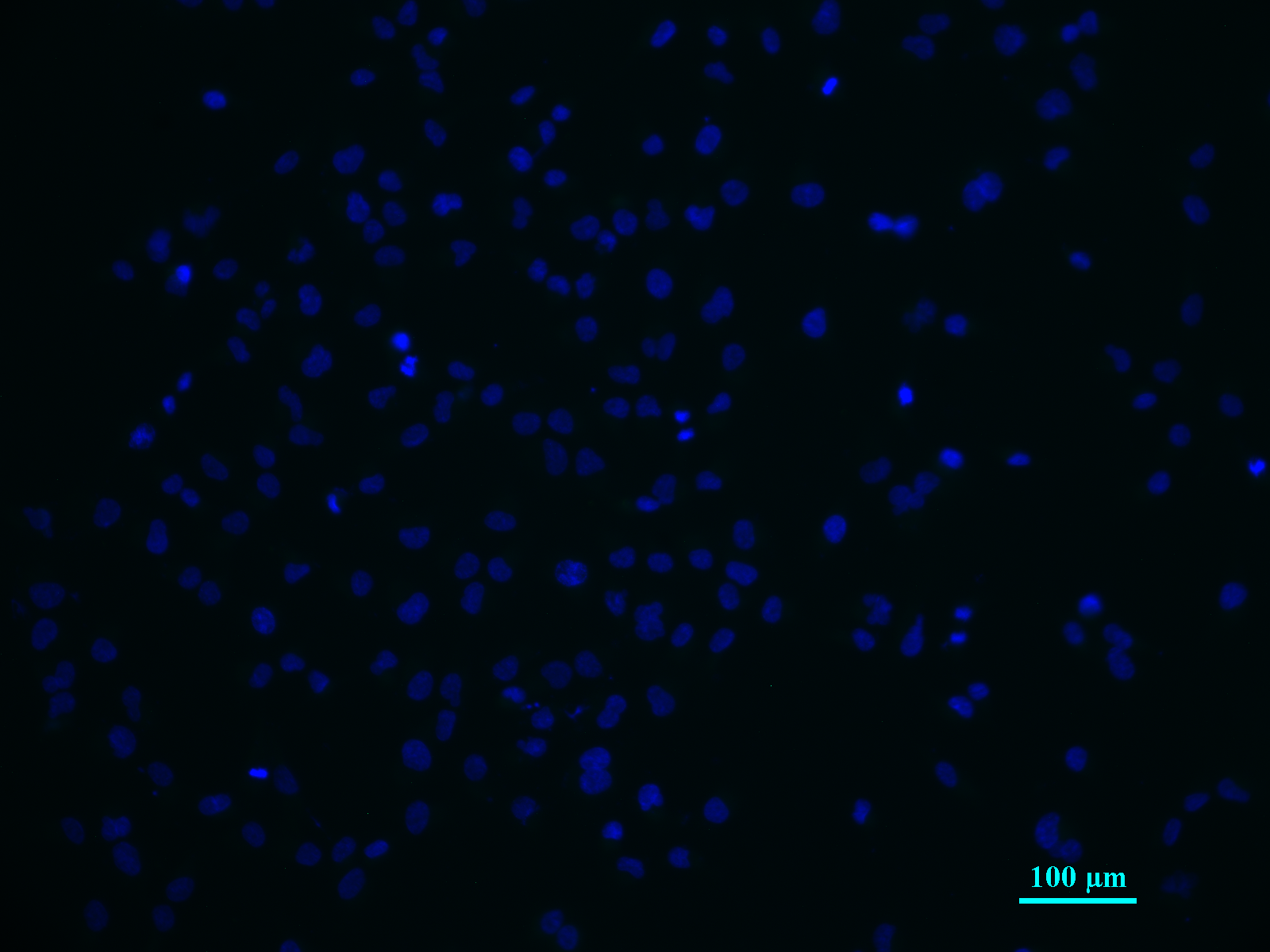

Supplement: Supplementary file 1 [file ijms-26-07956-s001.zip › immunofluorescenzaa MMP14/Figure 2A BCPAP CTRL -.tif]

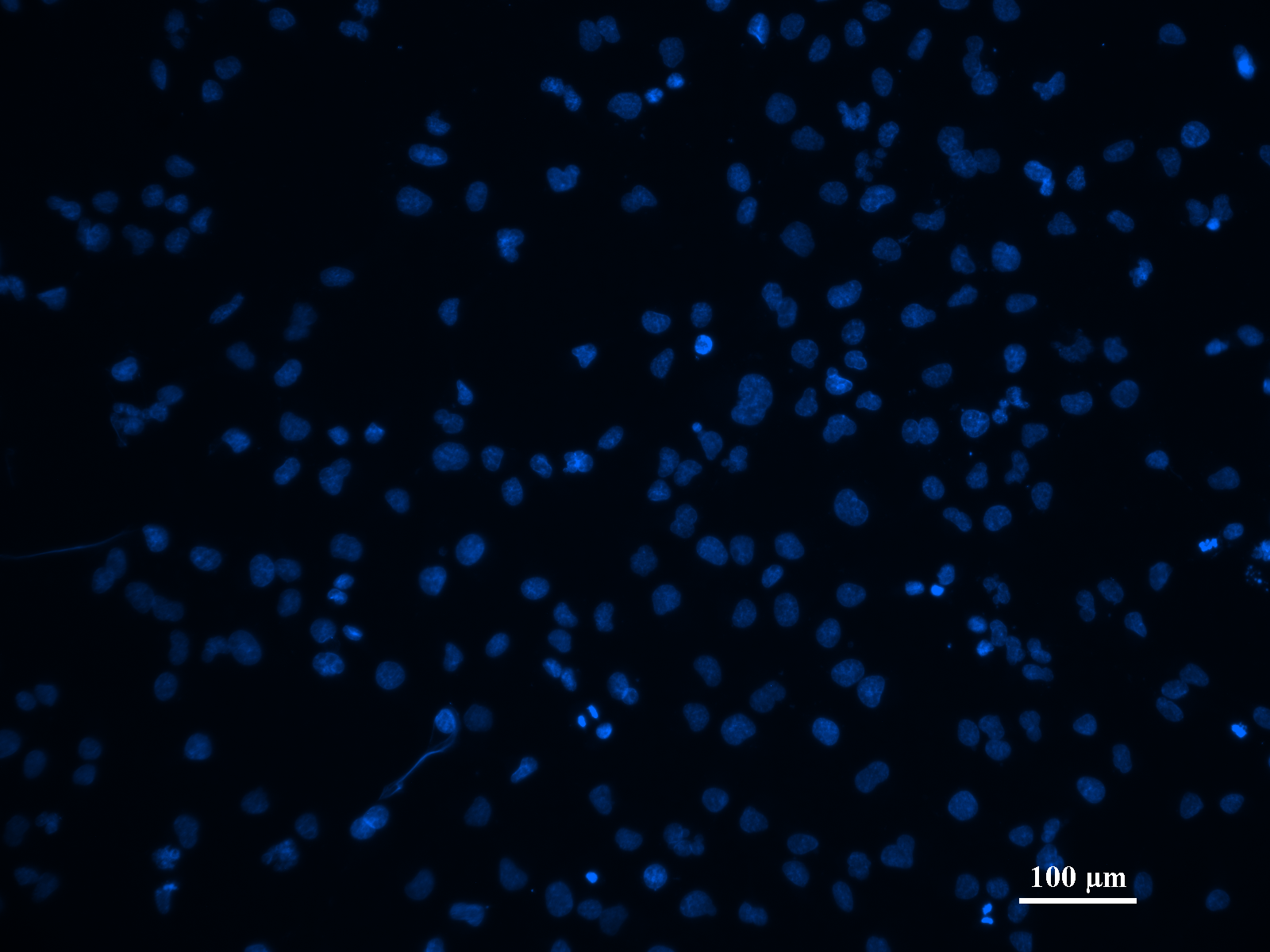

Supplement: Supplementary file 1 [file ijms-26-07956-s001.zip › immunofluorescenzaa MMP14/Figure 2A BCPAP dapi.tif]

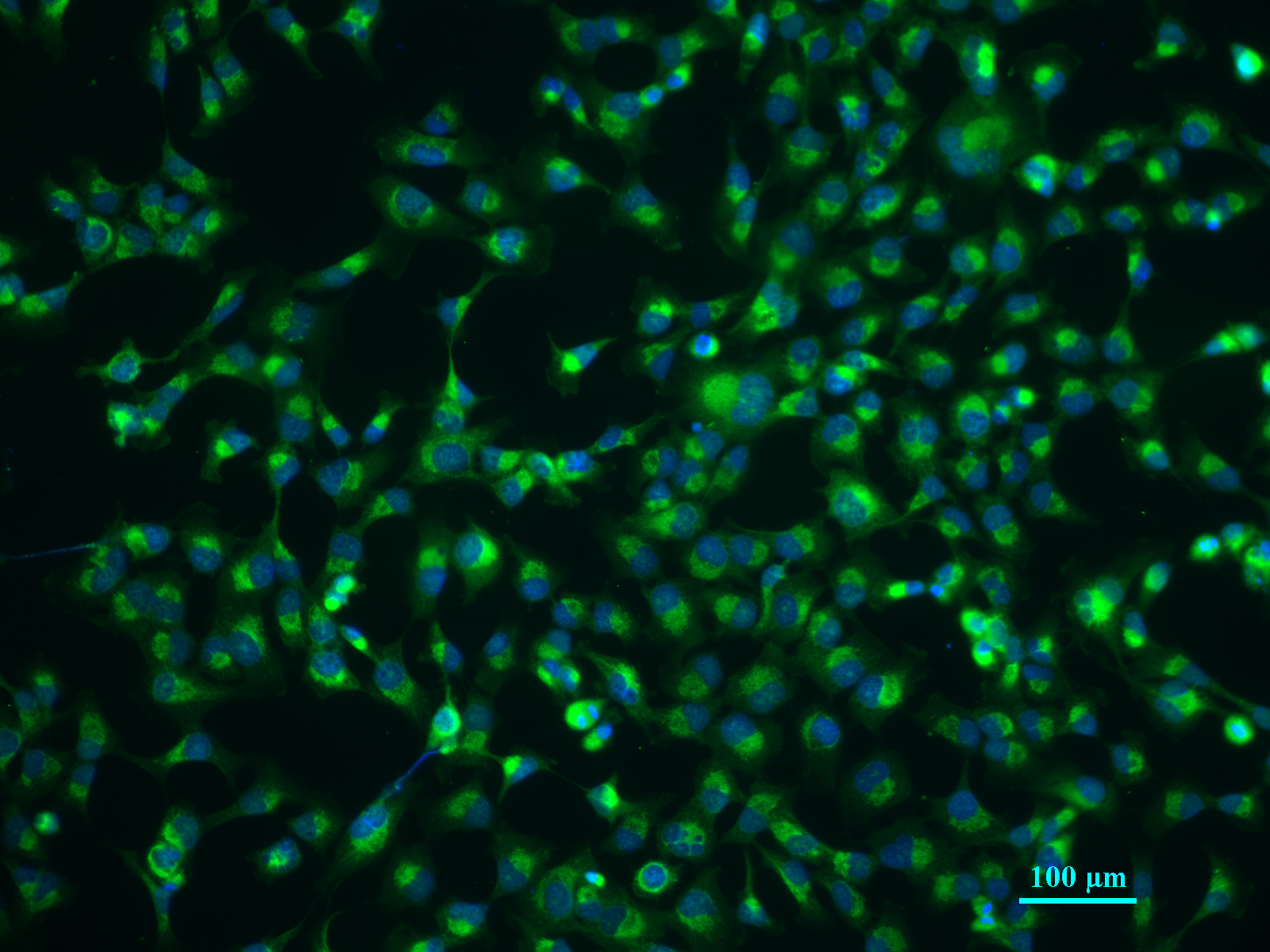

Supplement: Supplementary file 1 [file ijms-26-07956-s001.zip › immunofluorescenzaa MMP14/Figure 2A BCPAP merge.tif]

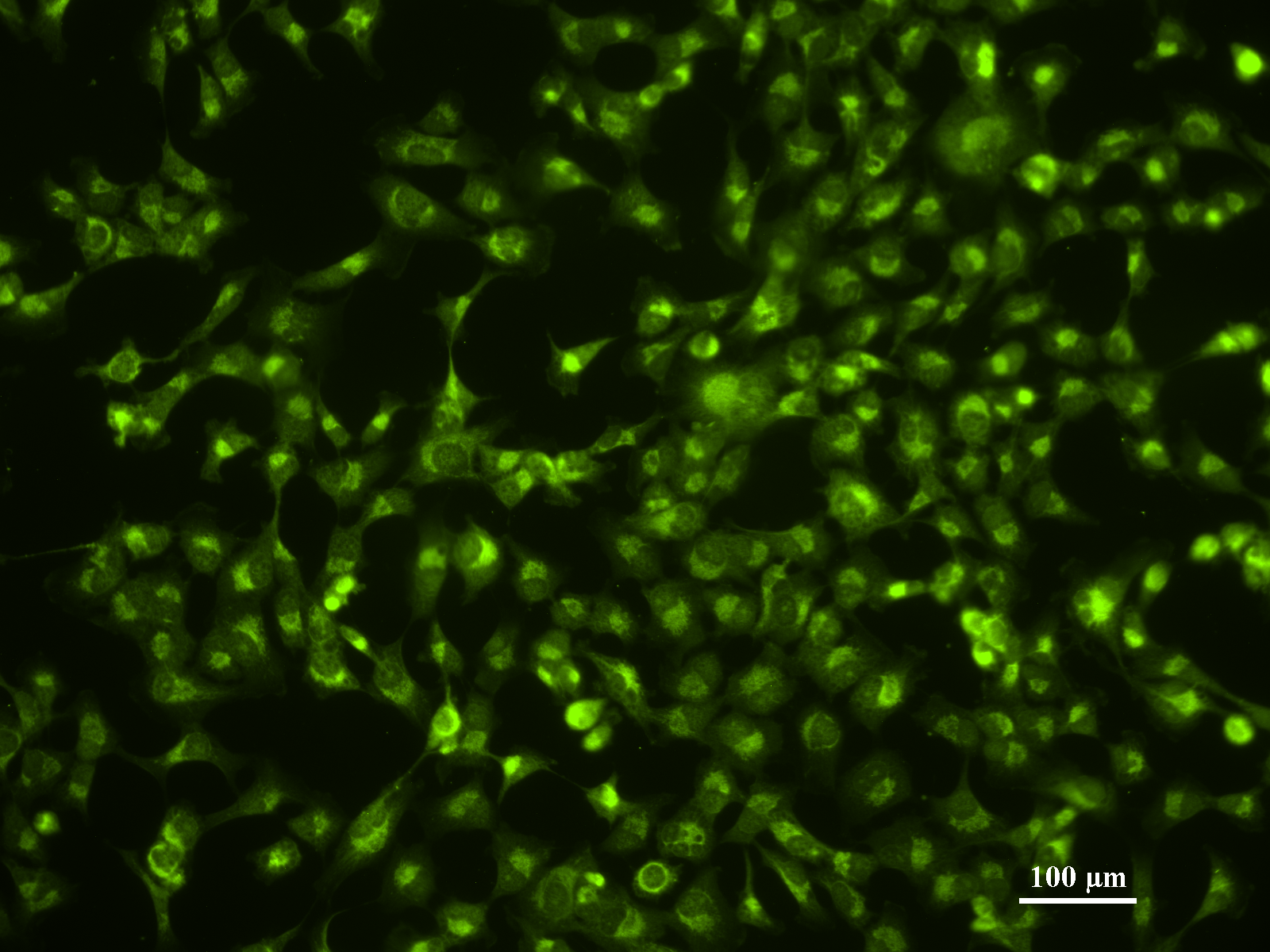

Supplement: Supplementary file 1 [file ijms-26-07956-s001.zip › immunofluorescenzaa MMP14/Figure 2A BCPAP MMP14.tif]

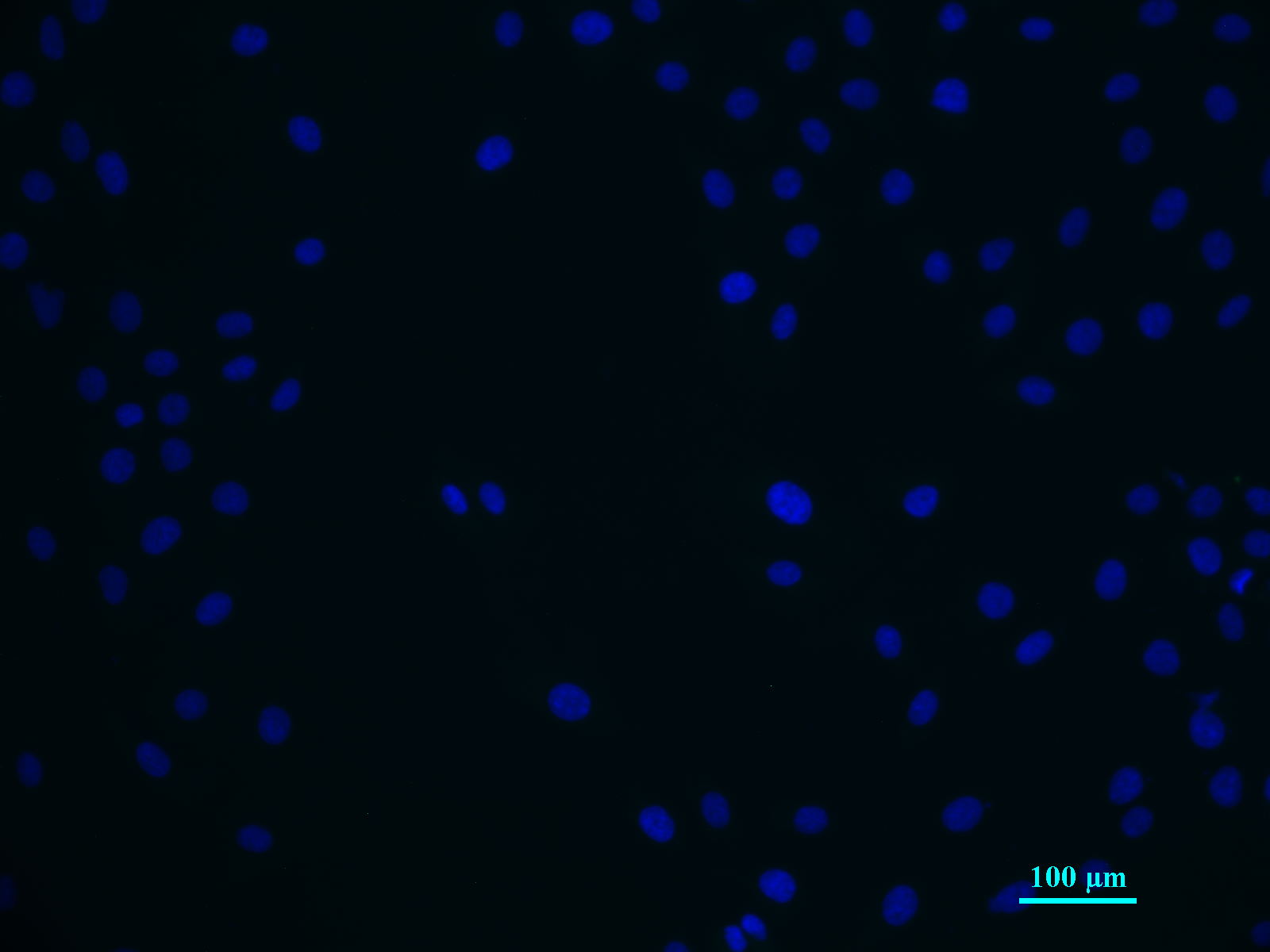

Supplement: Supplementary file 1 [file ijms-26-07956-s001.zip › immunofluorescenzaa MMP14/Figure 2A K1 CTRL -.tif]

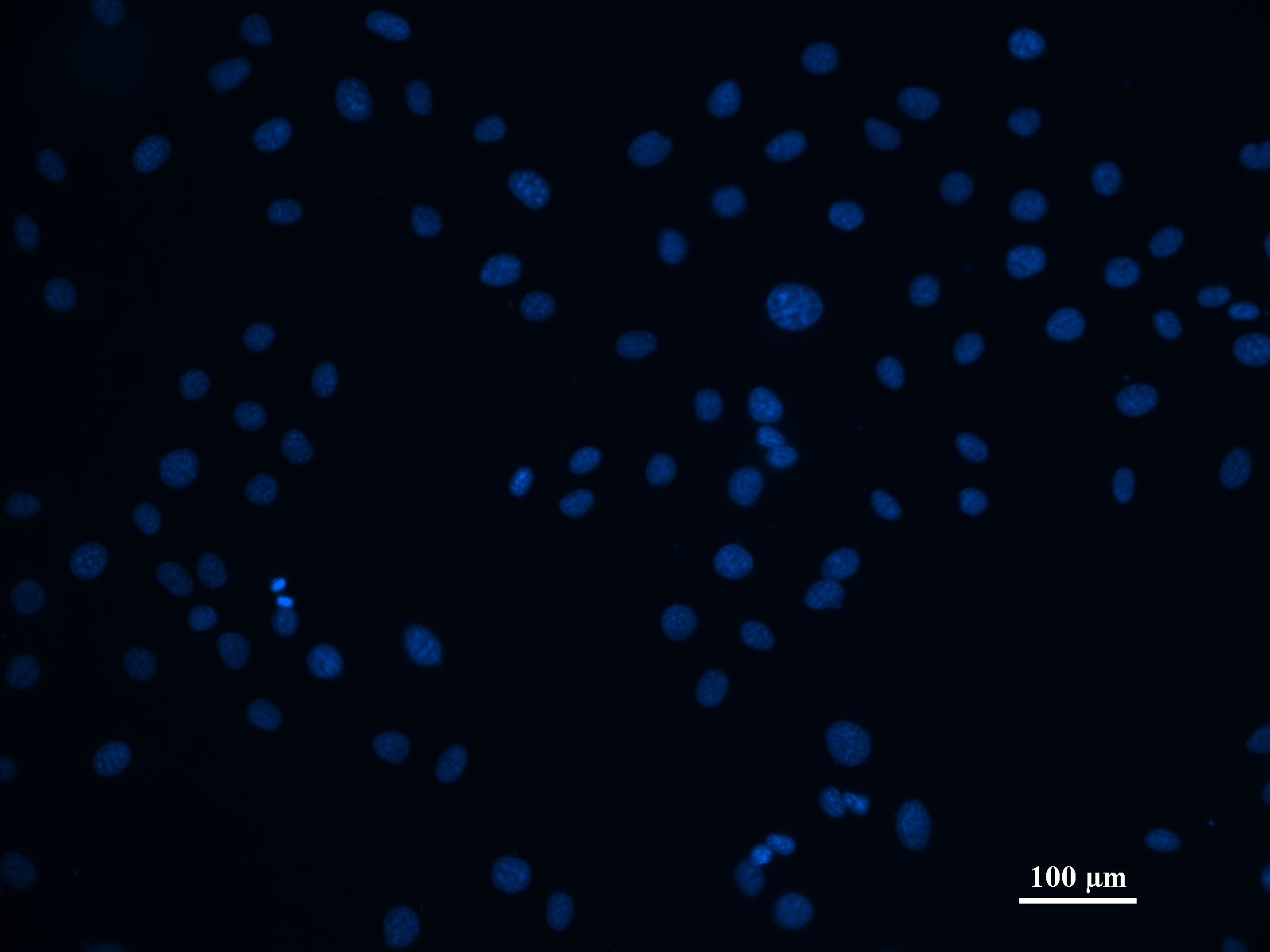

Supplement: Supplementary file 1 [file ijms-26-07956-s001.zip › immunofluorescenzaa MMP14/Figure 2A K1 dapi.tif]

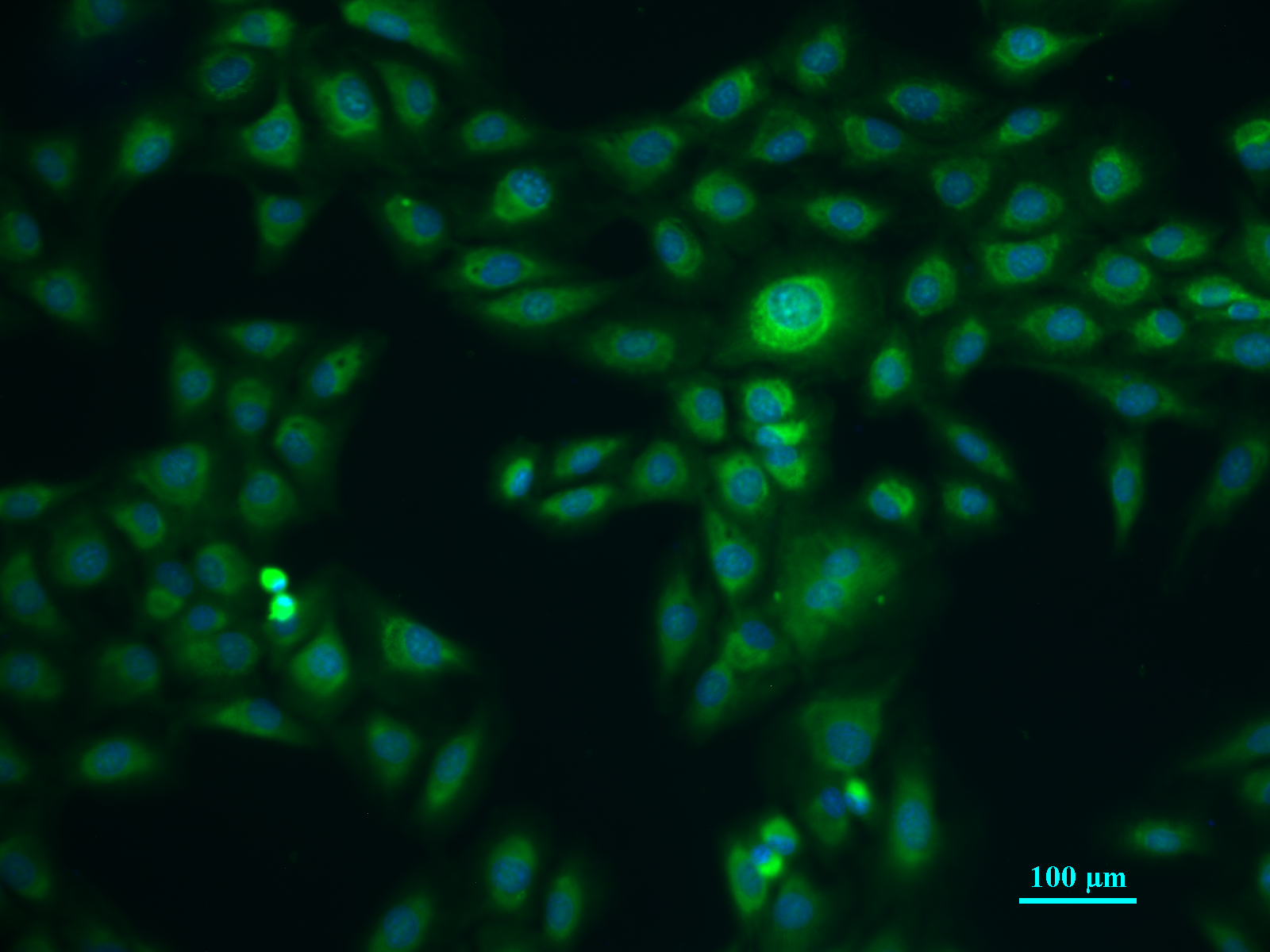

Supplement: Supplementary file 1 [file ijms-26-07956-s001.zip › immunofluorescenzaa MMP14/Figure 2A K1 merge.tif]

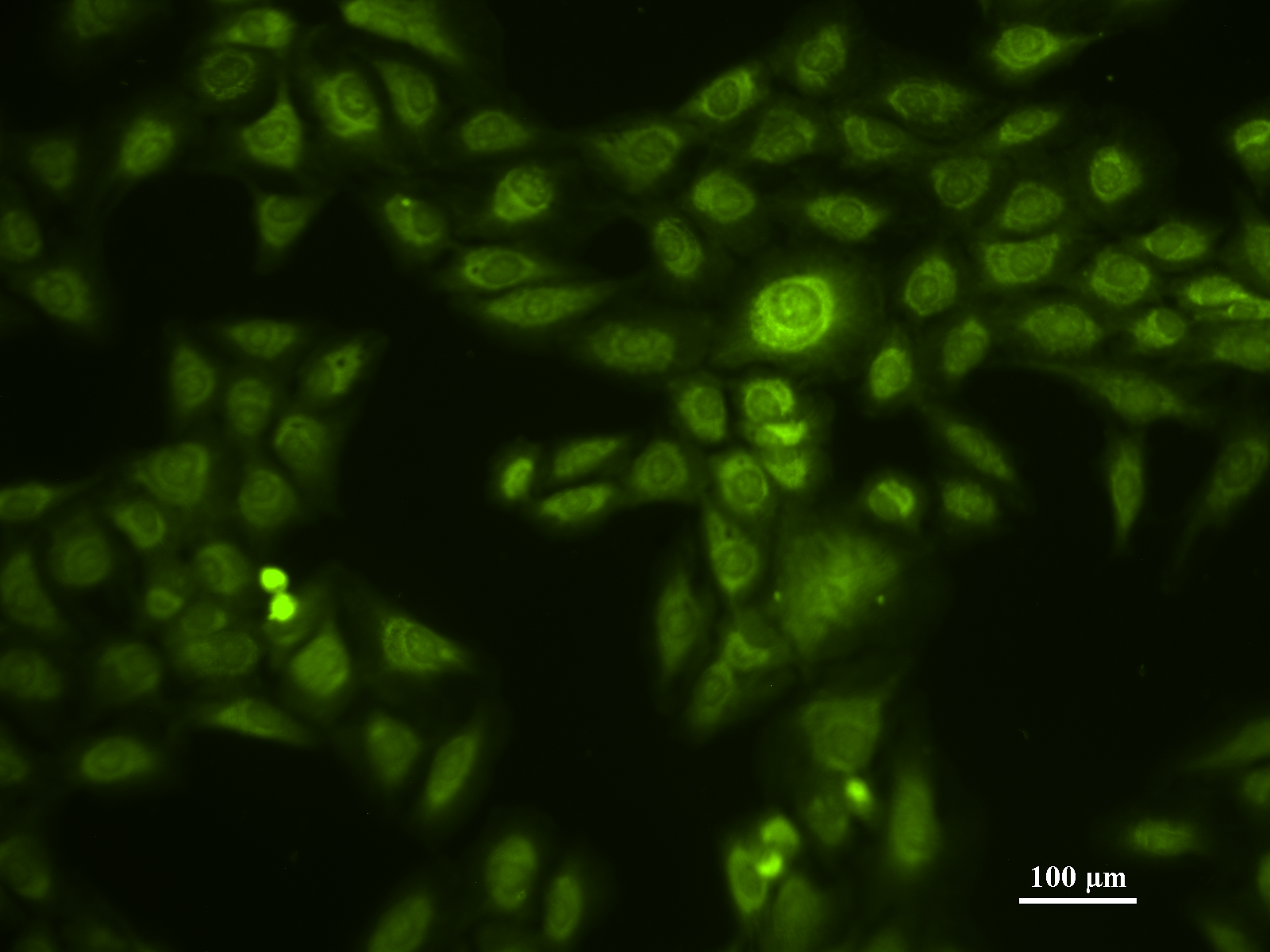

Supplement: Supplementary file 1 [file ijms-26-07956-s001.zip › immunofluorescenzaa MMP14/Figure 2A K1 MMP14.tif]

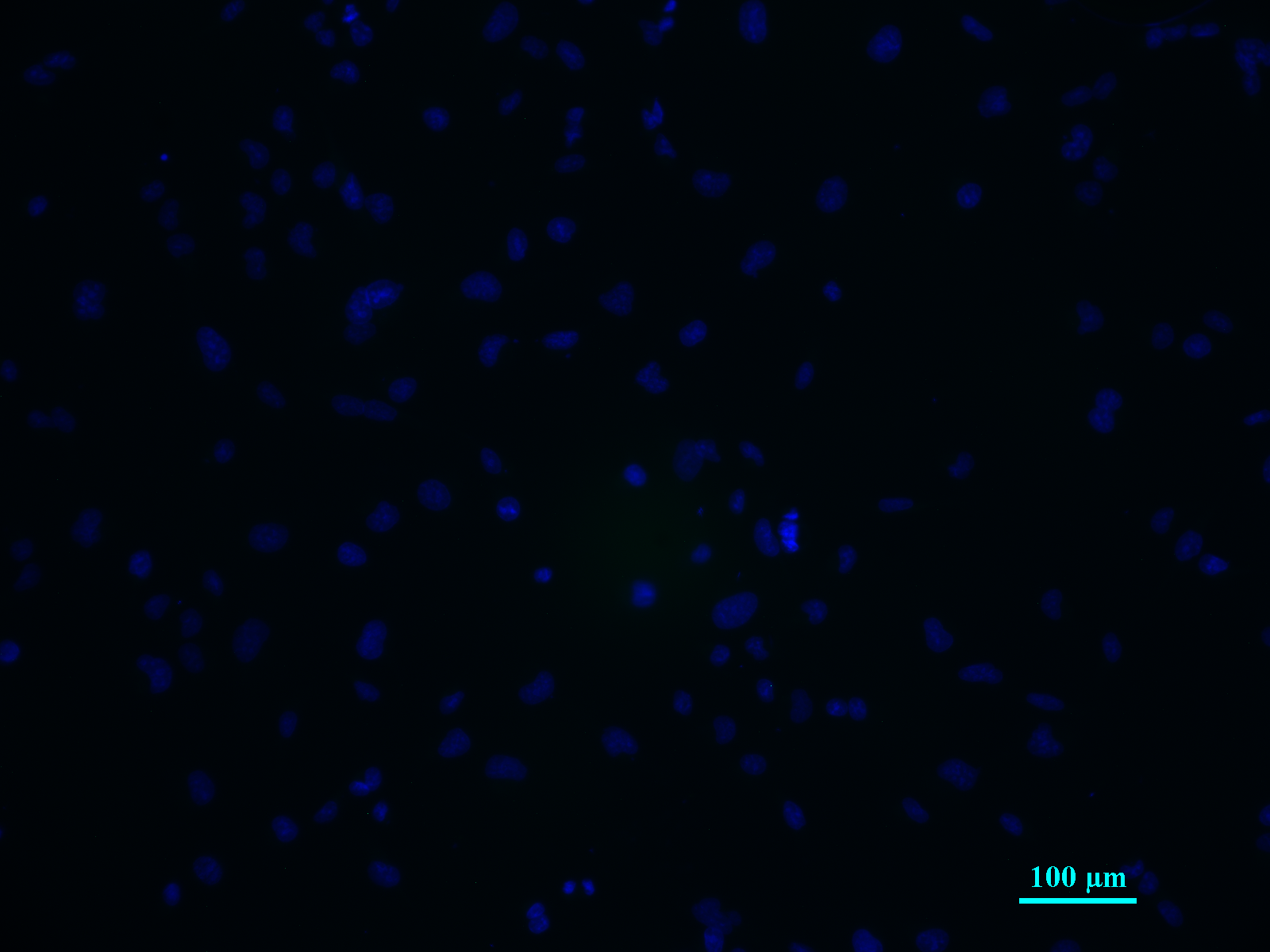

Supplement: Supplementary file 1 [file ijms-26-07956-s001.zip › immunofluorescenzaa MMP14/Figure 2A Nthy-ori-3.1 CTRL -.tif]

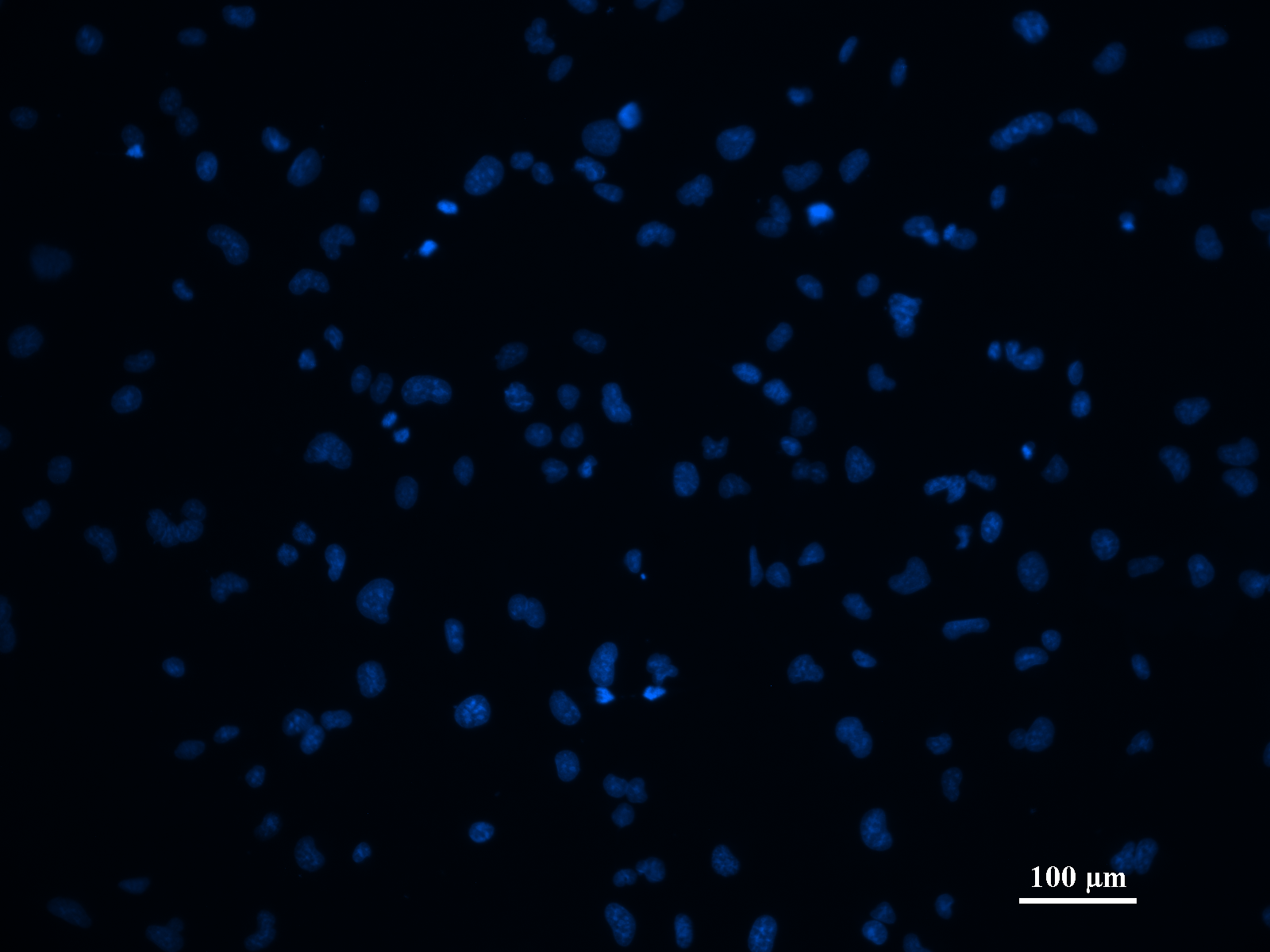

Supplement: Supplementary file 1 [file ijms-26-07956-s001.zip › immunofluorescenzaa MMP14/Figure 2A Nthy-ori-3.1 dapi.tif]

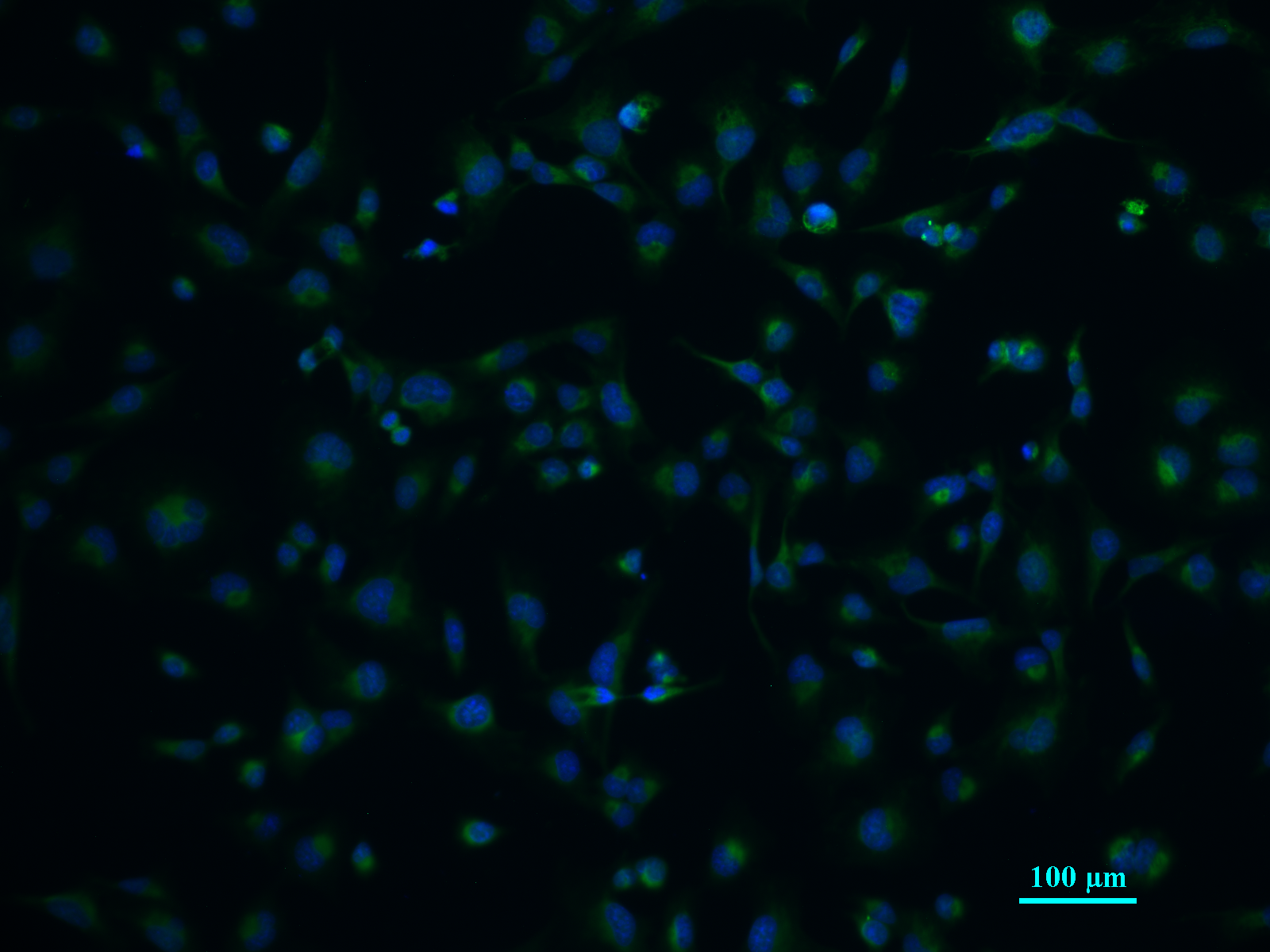

Supplement: Supplementary file 1 [file ijms-26-07956-s001.zip › immunofluorescenzaa MMP14/Figure 2A Nthy-ori-3.1 merge.tif]

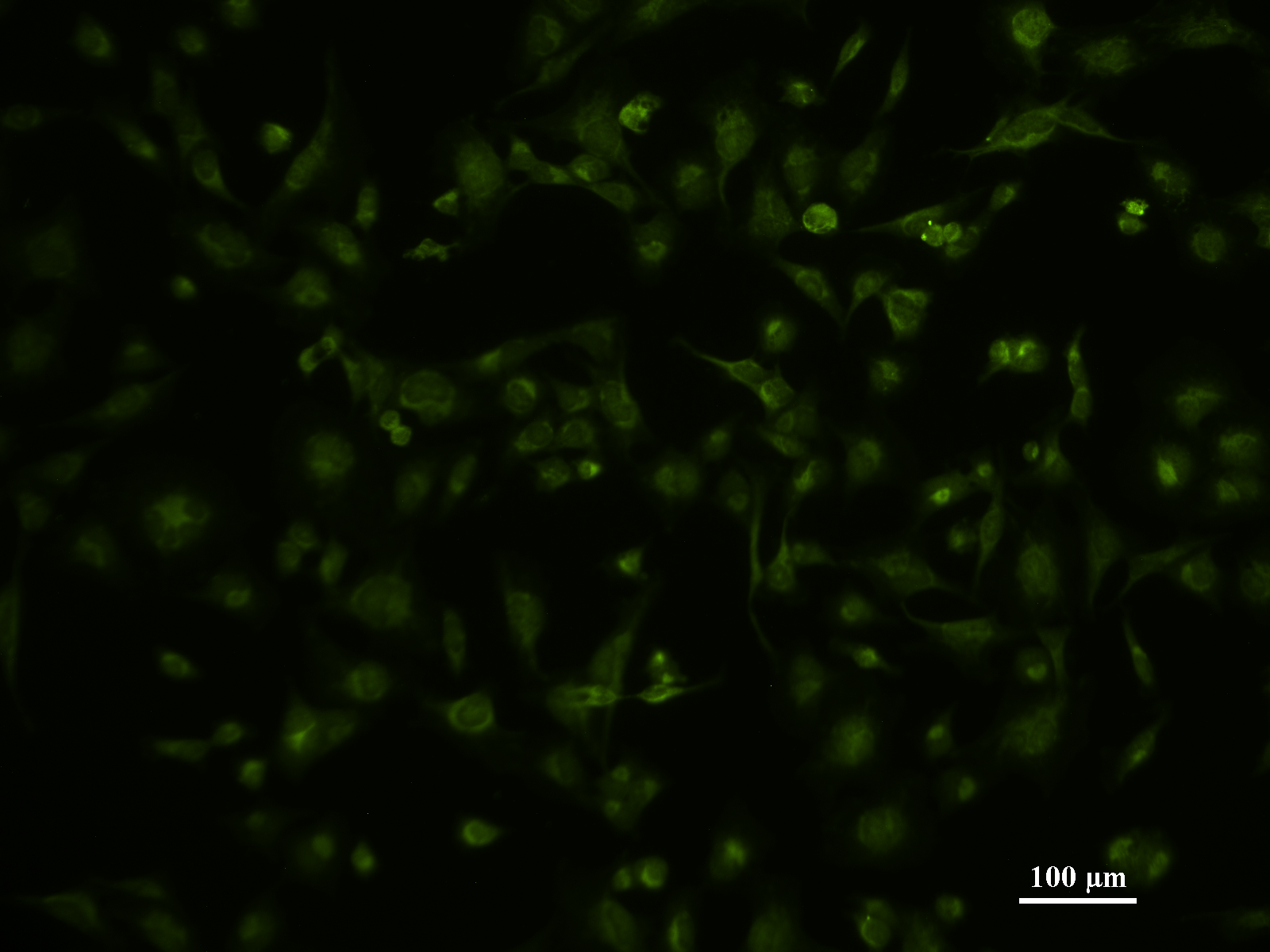

Supplement: Supplementary file 1 [file ijms-26-07956-s001.zip › immunofluorescenzaa MMP14/Figure 2A Nthy-ori-3.1 MMP14.tif]
